# Cutaneous Cell Therapy Manufacturing Timeframe Rationalization: Allogeneic Off-the-Freezer Fibroblasts for Dermo-Epidermal Combined Preparations (DE-FE002-SK2) in Burn Care

**DOI:** 10.3390/pharmaceutics15092334

**Published:** 2023-09-16

**Authors:** Xi Chen, Alexis Laurent, Zhifeng Liao, Sandra Jaccoud, Philippe Abdel-Sayed, Marjorie Flahaut, Corinne Scaletta, Wassim Raffoul, Lee Ann Applegate, Nathalie Hirt-Burri

**Affiliations:** 1 Plastic, Reconstructive and Hand Surgery Service, Lausanne University Hospital, University of Lausanne, CH-1011 Lausanne, Switzerland; xi.chen.1@unil.ch (X.C.); alexis.laurent@unil.ch (A.L.); liao.zhifeng@unil.ch (Z.L.); sandra.jaccoud@chuv.ch (S.J.); philippe.abdel-sayed@chuv.ch (P.A.-S.); marjorie.flahaut@chuv.ch (M.F.); corinne.scaletta@chuv.ch (C.S.); wassim.raffoul@chuv.ch (W.R.); 2 Manufacturing Department, TEC-PHARMA SA, CH-1038 Bercher, Switzerland; 3 Manufacturing Department, LAM Biotechnologies SA, CH-1066 Epalinges, Switzerland; 4 Laboratory of Biomechanical Orthopedics, Federal Polytechnic School of Lausanne, CH-1015 Lausanne, Switzerland; 5 STI School of Engineering, Federal Polytechnic School of Lausanne, CH-1015 Lausanne, Switzerland; 6 Lausanne Burn Center, Lausanne University Hospital, University of Lausanne, CH-1011 Lausanne, Switzerland; 7 Center for Applied Biotechnology and Molecular Medicine, University of Zurich, CH-8057 Zurich, Switzerland; 8 Oxford OSCAR Suzhou Center, Oxford University, Suzhou 215123, China

**Keywords:** autologous keratinocytes, burn center, cutaneous cell therapy, dermal template, dermo-epidermal grafts, early coverage solutions, FE002 dermal progenitor fibroblasts, manufacturing optimization, severe burns, standardized skin grafts

## Abstract

Autologous cell therapy manufacturing timeframes constitute bottlenecks in clinical management pathways of severe burn patients. While effective temporary wound coverings exist for high-TBSA burns, any means to shorten the time-to-treatment with cytotherapeutic skin grafts could provide substantial therapeutic benefits. This study aimed to establish proofs-of-concept for a novel combinational cytotherapeutic construct (autologous/allogeneic DE-FE002-SK2 full dermo-epidermal graft) designed for significant cutaneous cell therapy manufacturing timeframe rationalization. Process development was based on several decades (four for autologous protocols, three for allogeneic protocols) of in-house clinical experience in cutaneous cytotherapies. Clinical grade dermal progenitor fibroblasts (standardized FE002-SK2 cell source) were used as off-the-freezer substrates in novel autologous/allogeneic dermo-epidermal bilayer sheets. Under vitamin C stimulation, FE002-SK2 primary progenitor fibroblasts rapidly produced robust allogeneic dermal templates, allowing patient keratinocyte attachment in co-culture. Notably, FE002-SK2 primary progenitor fibroblasts significantly outperformed patient fibroblasts for collagen deposition. An ex vivo de-epidermalized dermis model was used to demonstrate the efficient DE-FE002-SK2 construct bio-adhesion properties. Importantly, the presented DE-FE002-SK2 manufacturing process decreased clinical lot production timeframes from 6–8 weeks (standard autologous combined cytotherapies) to 2–3 weeks. Overall, these findings bear the potential to significantly optimize burn patient clinical pathways (for rapid wound closure and enhanced tissue healing quality) by combining extensively clinically proven cutaneous cell-based technologies.

## 1. Introduction

Despite considerable constitutive regenerative potential, human skin is often incapable of managing serious burn injuries without exogenous interventions [1,2]. Thermal burns cause necrosis of the epidermis and of underlying tissues and structures. The extent of the related damage depends on the temperature to which the cutaneous cells and tissues are exposed and on the duration of said exposure [1]. The resulting high morbidity is intrinsically modulated by the wound depth and surface (i.e., burn degree) or by the severity of inhalation co-injuries [1,2]. Specifically, major burn victims are qualified upon initial presentation of ≥20% total body surface area (TBSA) lesions for adults and ≥10% TBSA lesions for pediatric patients [1,2,3]. Overall, severe burn patient care, remission, and rehabilitation are often long and painful [2,3].

Highly specialized therapeutic care centers are necessary for the effective management of severe burn victims [3]. With a yearly incidence of around 100 adult and pediatric burn cases, Switzerland is served by three specialized and multidisciplinary university hospital platforms (i.e., combined adult and pediatric pathway in Western Switzerland; separate centers in Eastern Switzerland) [3,4,5]. When considering the acute phase of burn victim care, patient resuscitation is followed by lesion characterization and escharotomies, as required (Figure S1). During the subsequent cardiorespiratory stabilization phase, the affected area is debrided, and a topical antiseptic (e.g., Ialugen Plus, Aquacel Ag) is applied [3]. Rapid wound coverage may be attained using appropriate bandages such as Mepitel, Polymem, Kaltostat, or DuoDerm. In severe cases, cadaver skin (i.e., human or porcine) or advanced coverage solutions (e.g., TransCyte, Lyphoderm, Apligraf, ReCell, OrCel, Epicel, Allox) can be deployed to prevent catastrophic fluid loss and to stimulate structural/functional restauration [6,7,8,9,10,11,12]. Thereafter, cutaneous stabilization is addressed by permanent wound closure using various surgical and/or grafting procedures [2,3,4,5,10].

From a cytotherapeutic viewpoint, burn wound stabilization and closure have been successfully performed using stratified autologous keratinocyte cultures (i.e., cultured epithelial autografts, CEA, Figure S2) [13,14,15,16]. In addition to half a century of available clinical hindsight, this approach is characterized by limited iatrogenesis related to autologous skin harvests, contrasting with split-thickness grafting [17,18,19,20,21,22,23,24]. While constituting a therapeutic breakthrough, classical CEA-based protocols are inherently technically limited by the in vitro graft manufacturing timeframes of 2–3 weeks [3,4,21]. Despite the commercial availability of various autologous keratinocyte preparation types (i.e., cell sheets or spray formats, e.g., EpicelT, ReCell), widespread clinical adoption has not yet been achieved. Notably, dependency towards the use of embryonic 3T3 murine feeder layers (i.e., for autologous keratinocyte culture) and the extensive manufacturing temporal constraints have not yet been satisfactorily addressed [25,26].

Furthermore, it has been reported for severe burn patients that CEA-treated cutaneous structures are often characterized by sub-optimal quality and by mechanical fragility due to the absence of a dermis to support the epidermal layer or due to poor graft integration [18]. To enhance the efficacy of the intervention, the tissue engineering technique has evolved to include a co-cultured basal dermal component (i.e., functionally stimulated patient fibroblasts), forming cultured dermo-epidermal autografts (CDEAs, Figure S3) [18,20,27]. However, the major technical drawback incurred by this combinational approach is the additionally extended manufacturing timeframe of 6–8 weeks for clinical grade CDEA production [28,29,30,31]. Notwithstanding, available clinical reports on CDEA use in burn care have confirmed the superior efficacy and enhanced tissular repair quality compared to CEA treatment [27,28,29,30,31,32]. Mechanistically, human dermal fibroblasts have notably been functionally characterized to aid in modeling collagen fibers and in secreting factors for epidermalization. Therefore, the presence of functional fibroblasts in modern cutaneous cytotherapies was considered necessary to promote epidermal outgrowth [33,34].

Clinically applied in the Lausanne Burn Center for over twenty years, CDEA constructs were shown to enhance the quality of skin repairs in burn victims compared to CEAs, themselves clinically used locally since 1985 [27]. However, the lack of an optimal early wound coverage solution for transient wound management prior to CDEA application was assessed as a major clinical bottleneck [5,10]. Therefore, in order to mitigate risks for the patient and to optimally prepare the wound bed before CDEA grafting, Integra membranes were locally used as temporary coverings [21,27]. As an alternative and bioactive early wound coverage solution, allogeneic progenitor biological bandages (PBBs) were developed and have been clinically applied in Lausanne since 2004 [35,36,37,38,39]. Designed and manufactured under the Swiss progenitor cell transplantation program, PBBs consist of banked allogeneic dermal progenitor fibroblasts (i.e., clinical grade FE002-SK2 cell source) formulated for topical delivery on resorbable equine collagen sheets (Figure S4, Table S1) [35,36,37,38,39]. Over two decades, viable allogeneic dermal progenitor fibroblasts have been cytotherapeutically applied on over 160 patients in Lausanne, where no safety-related concerns have been raised and an enhanced repair tissue quality was clearly evidenced [37,39]. Importantly, PBBs were found to optimally act as a “first cover“ solution in view of early wound bed preparation (i.e., biological stimulation over the first 10–12 days) for eventual autologous skin grafting [37]. In several reported clinical cases, the use of PBBs alone drastically reduced or even negated the need for subsequent skin grafting, CEA use, or CDEA use [36,37,38,39].

Based on the combined translational and clinical experience gained in the Lausanne Burn Center with CEAs, CDEAs, and PBBs, a new generation of autologous/allogeneic constructs was devised for significant manufacturing and clinical workflow rationalization. Therefore, the objective of this study consisted in the establishment of in vitro and ex vivo functional proofs-of-concept for the DE-FE002-SK2 dermo-epidermal construct based on allogeneic dermal progenitor fibroblasts and autologous keratinocytes. Clinical grade FE002-SK2 cells were firstly used as standardized off-the-freezer allogeneic substrates and were stimulated with vitamin C for rapid preparation of a collagen-rich dermal template [40]. Secondly, patient primary keratinocytes were co-cultured on the allogeneic dermal template to form a stratified epidermal layer. Importantly, the presented multiphasic process for DE-FE002-SK2 dermo-epidermal construct preparation decreased the cutaneous graft manufacturing timeframes from 6–8 weeks (i.e., standard CDEA protocol) to 2–3 weeks. Finally, an ex vivo de-epidermalized dermis (DED) model was used to functionally characterize the DE-FE002-SK2 construct and validate its applicability in a clinical setting. Overall, this work sets the technical basis for potentially significantly optimized burn patient clinical care using clinically proven and standardized biologicals with rationalized manufacturing resources.

## 2. Materials and Methods

### 2.1. Ethical Compliance of the Study

Obtention and use of patient primary cellular materials (i.e., primary fibroblast and keratinocyte cell types) followed the regulations of the Biobank of the Department of Musculoskeletal Medicine at the CHUV (Lausanne University Hospital, Lausanne, Switzerland). Biological materials and anonymous patient information materials were included in the biobank following patient consent documentation and protocol validation by the Vaud Cantonal Ethics Committee (University Hospital of Lausanne, Ethics Committee Protocol N°264/12). The clinical grade primary progenitor cell source used in the present study (i.e., FE002-SK2 primary progenitor fibroblasts) was established from the FE002 organ donation, as approved by the Vaud Cantonal Ethics Committee (University Hospital of Lausanne, Ethics Committee Protocol N°62/07). The FE002 organ donation was registered under a federal cell transplantation program (i.e., Swiss progenitor cell transplantation program). Appropriate material traceability and anonymity maintenance protocols were applied during the study.

### 2.2. Materials and Consumables Used for the Study

The reagents and consumables which were used in this study are listed hereafter: purified water, PBS buffer, and NaCl 0.9% solutions (Bichsel, Unterseen, Switzerland); high-glucose DMEM cell culture medium, L-glutamine, D-PBS, TrypLE dissociation reagent, MTT, and antibodies (Thermo Fisher Scientific, Waltham, MA, USA); penicillin-streptomycin and trypsin (Life Technologies, Paisley, UK); CnT-PR medium (CellnTec, Bern, Switzerland); C-Chip Neubauer hemocytometers (NanoEntek, Seoul, Republic of Korea); ethanol and HCl (Chemie Brunschwig, Basel, Switzerland); methanol (Fluka, Buchs, Switzerland); EDTA and gentamycin (Lausanne University Hospital, Lausanne, Switzerland); mitomycin C (Medac Pharma, Chicago, IL, USA); cholera toxin (Lubio Science, Zurich, Switzerland); hydrocortisone (Pfizer, New York, NY, USA); insulin (Novo Nordisk Pharma, Bagsværd, Danemark); Millipore Stericup with 0.22 μm pores, milliQ water, Ham’s F12 medium, EGF, vitamin C, picric acid, sodium hydroxide, sodium chloride, Trypan Blue solution, and FBS (Merck, Darmstadt, Germany); formalin-buffered solution, dispase, paraffin, H&E stain, xylene, and H_2_O_2_ (Sigma Aldrich, Buchs, Switzerland); Sirius Red solution and antibodies (Abcam, Cambridge, UK); antibodies (Vector Laboratories, Newark, CA, USA); a ChromoMap DAB kit (Roche Diagnostics, Rotkreuz, Switzerland); rat tail collagen and agar (Corning Life Sciences, Tewksbury, MA, USA); cell culture vessels and plastic assay surfaces (Greiner BioOne, Frickenhausen, Germany; Corning, New York, NY, USA; and TPP Techno Plastic Products, Trasadingen, Switzerland); and Vaseline gauze (Smith and Nephew, Watford, UK).

### 2.3. Equipment Used for the Study

Component weighing was performed on a laboratory scale (Ohaus, Parsippany, NJ, USA). Sample centrifugation was performed on a Sorvall Legend Micro 21R microcentrifuge (Thermo Fisher Scientific, Waltham, MA, USA). Absorbance measurements were performed on a Varioskan LUX multimode plate reader (Thermo Fisher Scientific, Waltham, MA, USA). Absorbance data were analyzed using the Skanit-RE software v5.0 (Thermo Fisher Scientific, Waltham, MA, USA). Immunohistology sample preparation was performed using a Ventana Discovery ULTRA system (Roche Diagnostics, Rotkreuz, Switzerland). Immunohistochemistry imaging was performed on an inverted IX81 fluorescence microscope (Olympus, Tokyo, Japan). Macroscopic imaging was performed on an iPhone 12 (Apple, Cupertino, CA, USA).

### 2.4. Primary Cell Sourcing and Cellular Raw Material Manufacture

Patient primary fibroblasts and keratinocytes were isolated and cultured from skin tissue designated as medical waste following routine abdominoplasties at the Lausanne University Hospital. At reception, each skin biopsy was washed several times in PBS with 1% penicillin–streptomycin to remove the residual blood cells. Subcutaneous fat was completely ablated using a scalpel. Then, the skin biopsies were incubated in DMEM with 10 mg/mL dispase overnight at 4 °C and then in 0.05% trypsin–EDTA for 30 min at 37 °C, after which the dermis was mechanically separated from the epidermis using forceps.

For primary keratinocyte isolation by enzymatic digestion, the epidermis was cut into small fragments and was transferred to a tube containing EDTA 0.02% and trypsin 0.25% in 1:1 proportion. The digestion tubes were incubated for 30 min at 37 °C on a rotational shaker. This step was repeated at least twice to obtain a maximal cell yield. Then, keratinocytes were enumerated and were seeded in vitro at a density of 2–3 × 10^4^ cells/cm^2^. The cells were either seeded in CnT-PR medium and incubated at 37 °C under 5% CO_2_ or they were cultured on a feeder layer of proprietary 3T3-J2 mouse fibroblasts. The 3T3-J2 feeder fibroblasts were inactivated for 2 h using 4 µg/mL mitomycin C. In the feeder layer group, the keratinocyte proliferation medium was composed of DMEM and Ham’s F12 with a 3:1 proportion, with 20 µg/mL gentamycin, 0.14 nM cholera toxin, 400 ng/mL hydrocortisone, 8.3 ng/mL EGF, 832.2 µM L-glutamine, 0.12 U/mL insulin, and 10% FBS. All the cultures were maintained in humidified incubators at 37 °C with 5% CO_2_ and the cell proliferation medium was exchanged three times per week. Primary keratinocytes were serially expanded in vitro and were used for experiments at passage levels 3–6. The cells were cryopreserved in a solution of 50% DMEM, 40% FBS, and 10% DMSO at a cellular density of 10^6^ viable cells/vial.

For primary fibroblast isolation by explanting, the dermis was cut into small fragments and was transferred to a tissue culture dish. The tissue fragments were minced and placed within a checkboard pattern created on the culture surface by mechanical scoring. The fibroblast proliferation medium was composed of high-glucose DMEM supplemented with 2 mM L-glutamine and 10% FBS. The cultures were maintained in humidified incubators at 37 °C with 5% CO_2_ and the cell proliferation medium was exchanged twice per week. Once the cells reached 50–70% confluency, they were transferred and expanded in culture flasks, with medium exchange procedures performed twice per week. Primary fibroblasts were serially expanded in vitro and were used for experiments at passage levels 3–6. The cells were cryopreserved as described hereabove.

For primary dermal progenitor fibroblast use, cryopreserved cellular materials were procured under the Swiss progenitor cell transplantation program. Clinical grade FE002-SK2 primary progenitor fibroblast vials were used as cellular starting materials and were serially expanded in vitro. The cells were seeded at viable densities of 1.5 × 10^3^, 3 × 10^3^, 5 × 10^3^, and 20 × 10^3^ cells/cm^2^ and were maintained in culture until the monolayers attained confluency. The progenitor fibroblast proliferation medium was composed of high-glucose DMEM supplemented with 2 mM L-glutamine and 10% FBS. The cultures were maintained in humidified incubators at 37 °C with 5% CO_2_ and the cell proliferation medium was exchanged twice per week. Primary progenitor fibroblasts were serially expanded in vitro and were used for experiments at passage levels 5–12. The cells were cryopreserved as described hereabove.

### 2.5. Dermal Template Preparation: In Vitro Collagen Synthesis Induction Conditions

In order to prepare the basal component of standard CDEAs or of DE-FE002-SK2 constructs, primary fibroblasts were used to synthesize extracellular matrix components (e.g., collagens). For the autologous group, primary patient fibroblasts were used according to the standard CDEA manufacturing protocols [3]. For the allogeneic group, clinical grade FE002-SK2 primary progenitor fibroblasts were used following the same protocol. Briefly, confluent fibroblast cultures were harvested and the cells were seeded in 12-well culture plates at a viable density of 3 × 10^3^ cells/cm^2^. The cultures were maintained as described hereabove. When the cells reached 100% confluency, the fibroblast proliferation medium was exchanged for fibroblast induction medium supplemented with 10^−4^ M vitamin C to induce collagen production. The induction medium was replaced every 2 days for 1 week. Then, the treated cell monolayers were washed with 1× PBS, fixed with −20 °C methanol for 10 min, rinsed 3 times with 1× PBS, and stored at −80 °C until further use.

### 2.6. Allogeneic Dermal Template Preparation: In Vitro Manufacturing Optimization

As global manufacturing timeframe rationalization constituted the main objective of this study, specific technical optimization work aimed to reduce the time necessary for allogeneic dermal template preparation for the DE-FE002-SK2 constructs to a minimum. Specific process design elements were taken into consideration, such as cellular material availability, GMP manufacturing suite occupation, and targeted production timelines [3]. Within the allogeneic protocol, cryopreserved vials of FE002-SK2 primary progenitor fibroblasts were thawed and used at various cell seeding densities (i.e., 1.5–20 × 10^3^ cells/cm^2^) to determine optimal and condensed culture technical specifications (i.e., shortest culture time with sparing use of cell stocks). The cultures were otherwise maintained as described hereabove. The time to confluency was monitored for each seeding cellular density group. Operator assessments were independently performed by 3 experienced cellular biologists (i.e., trained in the development and release of cutaneous grafts produced for severe burn patients) in standardized blind experimental set-ups.

### 2.7. Dermal Template Characterization Assay: Sirius Red Staining and Collagen Quantification

In order to quantitatively and comparatively assess the production of collagens by the prepared dermal templates, Sirius Red staining and subsequent quantification were performed at various timepoints of the primary fibroblast induction phase (i.e., after 0, 2, 4, and 7 days). To prepare a standard curve, serial dilutions of rat tail collagen (i.e., at 3200, 1600, 800, 400, 200, 100, 50, 25, 12.5, 6.25, and 0 µg/mL) were performed in 1× PBS. Equal 150 µL volumes of collagen standard were dispensed in triplicate in a 96-well microplate. The plates were incubated at 37 °C overnight to evaporate the liquid phase. Each well was then gently rinsed 4 times with milliQ water. The collagen standards were stained with 150 µL of 0.1% Sirius Red in 1.3% picric acid aqueous solution for 1 h at ambient temperature and under agitation. Macroscopic and microscopic imaging were performed. For Sirius Red stain quantification, 150 µL of 0.1 M NaOH was added to the wells and the plates were incubated for 30 min at ambient temperature under agitation. The samples were then transferred to a new 96-well microplate and absorbance measurements were performed at a wavelength of 560 nm. For the analysis of the prepared dermal template samples, the corresponding 12-well plates were thawed at ambient temperature. The same Sirius Red staining and quantification steps were performed as described hereabove for the reference standards. The data were analyzed using the Skanit-RE software with a linear regression curve.

### 2.8. Combined DE-FE002-SK2 Construct Manufacturing Process

In order to prepare DE-FE002-SK2 dermo-epidermal constructs, FE002-SK2 primary progenitor fibroblasts were seeded in 6-well plates at a viable density of 3 × 10^3^ cells/cm^2^ and the cultures were maintained with medium exchanges performed twice weekly. Once confluency was reached after one week of culture, the cells were treated with fibroblast induction medium, which was exchanged every 2 days for one week. Primary patient keratinocytes were grown in parallel in CnT-PR medium or on 3T3-J2 feeder layers with keratinocyte proliferation medium. Upon reaching 60–80% confluency, the keratinocytes were enzymatically harvested to form a cell suspension and were transferred by cell seeding on top of the induced fibroblasts (i.e., 5–10 × 10^5^ keratinocytes/well). Then, the fibroblast–keratinocyte co-cultures were maintained for one week in keratinocyte proliferation medium.

At the end of the bilayer construct manufacturing phase, a part of the obtained samples was transferred onto a Vaseline gauze and was then deposited on top of a de-epidermalized dermis model (DED, see Section 2.10 for a process description of model preparation/validation) to mimic burned skin treatment. The other part of the obtained samples was washed in 1× PBS and was fixed with formalin-buffered solution for 15 min. Then, the sheets were gently detached from the wells with a brush, and 1% agar was poured in the wells. The samples were conserved at 4 °C for 3 days before being embedded in paraffin for histology assays.

In order to prepare epidermal constructs as controls, the standard CEA manufacturing protocol was used [3]. Briefly, primary patient keratinocytes were cultured on 3T3-J2 feeder layers until reaching hyperconfluency. Then, the stratified keratinocyte sheets were transferred onto a Vaseline gauze and were used for the DED or the histology assays.

### 2.9. Combined DE-FE002-SK2 Construct Structural Characterization: Histology and Immunohistochemistry Assays

In order to process the obtained constructs for histological analysis, a standard protocol was used. Briefly, the tissues were embedded in paraffin wax blocks and were sectioned to 7 μm in thickness with a microtome before being placed on glass slides. The slides were stained with hematoxylin and eosin (H&E) and Haris hematoxylin.

P63 immunohistochemistry was performed on the samples to assess the presence of keratinocytes in the different constructs. Therefore, the embedded sample sections were deparaffinized in xylene (i.e., twice for 10 min) and were sequentially passed through 100% ethanol (i.e., twice for 10 min), 90% ethanol (i.e., once for 10 min), and 74% ethanol (i.e., once for 10 min). Then, a 30 min incubation step in H_2_O_2_ (i.e., 10% in 1× PBS) was performed to block endogenous peroxidase activity. Subsequently, the sections were washed and then incubated with 2.5% horse serum for 1 h. The slides were then incubated overnight at 4 °C with a rabbit anti-p63 antibody (i.e., 1:2000 dilution, N°ab124762, Abcam) for human keratinocyte visualization. The following day, after the washing steps, the appropriate secondary antibody (i.e., horse anti-rabbit) was added to the slides and the samples were incubated for 1 h. The revelation step using ImmPact DAB was performed under the microscope for less than 2 min. Following immunohistochemical staining, the slides were counterstained with fastRed and mounted on resin with glass coverslips. The negative controls were obtained by omitting the addition of the primary antibodies.

Ki67 immunohistochemistry was performed on the samples to assess the presence of proliferating cells in the different constructs. Detection of cell proliferation was performed using a rabbit α-Ki67 antibody (i.e., 1:100 dilution, SP6, N°MA5-14520, Thermo Fisher Scientific) and the fully automated Ventana Discovery ULTRA system (Roche Diagnostics, Rotkreuz, Switzerland). Briefly, dewaxed and rehydrated paraffin sections were pre-treated with heat using standard conditions (40 min) in the CC1 solution. The samples were incubated with the primary antibodies at 37 °C for 1 h. After incubation with a secondary rabbit HRP antibody (i.e., N°MP 7401, Vector Laboratories), chromogenic revelation was performed with a ChromoMap DAB kit. The sections were counterstained with Harris hematoxylin and permanently mounted. Ki67 immunohistochemistry was performed at the Histology Core Facility of the Swiss Federal Institute of Technology in Lausanne (EPFL, Lausanne, Switzerland).

### 2.10. Combined DE-FE002-SK2 Construct Functional Characterization: Ex Vivo De-Epidermalized Dermis Model

The protocol for establishing the ex vivo DED model was adapted from MacNeil et al. [41]. Briefly, the DED model was prepared by cutting abdominoplasty skin sections into 1–2 cm^2^ pieces, which were then incubated at 37 °C for at least 24 h and up to 72 h in 1 M NaCl, where the duration of the incubation step was dependent on the treated sample. The incubation step in NaCl (i.e., 24 h in general) was sufficient to enable epidermis detachment, but without causing damage or destruction to the dermal component. The epidermis was delicately removed from the dermis using forceps to obtain a decellularized, de-epidermalized dermis (DED) section. The DED samples were washed several times with 1× PBS and were stored at 4 °C until use. For validation of the DED model (i.e., to verify the absence of keratinocytes on the dermal component following NaCl incubation), the experimental materials were qualified using histology, immunohistochemistry, and MTT controls (i.e., following treatment and after a recovery period in culture medium), for general exclusion of the presence of metabolically active cells and specific exclusion of the presence of keratinocytes on the dermal structures.

For the assays, DED samples were transferred into 12-well plates with 1 mL of keratinocyte proliferation medium and were incubated at 37 °C under 5% CO_2_ for 24 h. Then, the cultured DE-FE002-SK2 constructs (i.e., on Vaseline gauze) were transferred to the top of the DED sections and the plates were incubated for one week. The culture medium was exchanged twice weekly. After one week, a part of the samples was fixed in formalin for 24 h at ambient temperature, rinsed with 1× PBS, and placed in 70% ethanol at 4 °C until paraffin inclusion. Another part of the samples was stained with MTT to assess cellular viability and homogeneity. Therefore, DED samples were stained with 0.5 mg/mL MTT in PBS for 2 h at 37 °C and then washed twice with PBS.

Control samples were prepared using different combinations of cultured cells (i.e., FE002-SK2 fibroblasts and patient keratinocytes), which were deposited on the DED model. Therefore, 8-mm diameter glass inserts were placed on the surface of the DED sections and 100 µL of cell suspensions in proliferation medium was dispensed in the insert. The plates were incubated for 3 days to enable cellular attachment. Then, the glass inserts were removed and the DED samples were deposited on a plastic grid to constitute an air–liquid interface.

### 2.11. Statistical Analyses and Data Presentation

The presented experiments were performed in triplicate and using three experimental repetitions unless specified otherwise. For statistical comparison of average values from two sets of data, a paired Student’s *t*-test was applied after appropriate evaluation of the normal distribution of the data. For a statistical comparison of values from multiple quantitative datasets from experiments where multiple variables applied, a one-way ANOVA test or a two-way repeated measures ANOVA test was performed and was followed by a post hoc Tukey’s multiple comparison test. A *p*-value < 0.05 was retained as a base for statistical significance determination. The calculations were performed using Microsoft Excel (Microsoft Corporation, Redmond, WA, USA) and GraphPad Prism version 8.0.2 (GraphPad Software, San Diego, CA, USA). Data were presented using Microsoft PowerPoint and GraphPad Prism version 8.0.2.

## 3. Results

### 3.1. FE002-SK2 Primary Progenitor Fibroblasts Are Functionally Superior to Patient Fibroblasts for In Vitro Collagen Synthesis

**Figure 1:**
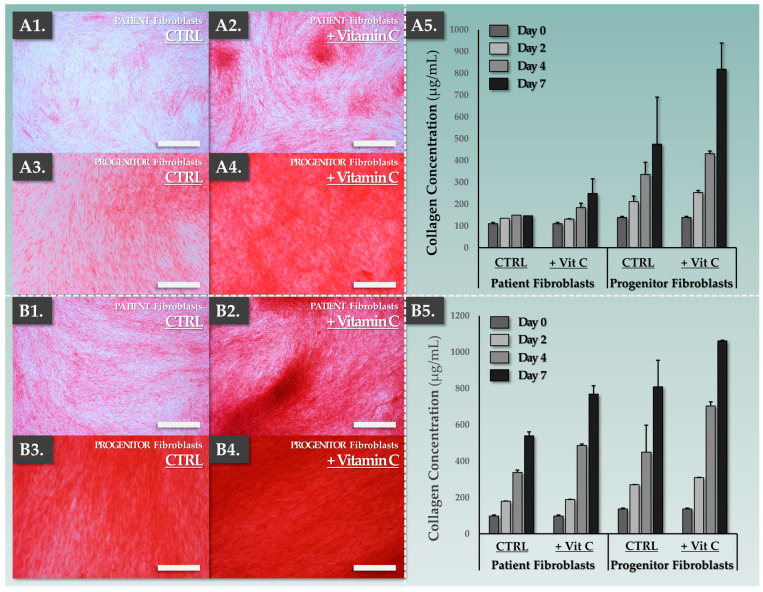
In vitro comparative functional characterization of primary patient fibroblasts and FE002-SK2 primary progenitor fibroblasts. (**A1**–**A4**) Endpoint imaging of Sirius Red staining for both primary fibroblast types following 7 days of vitamin C induction in fibroblast proliferation medium. Scale bars = 400 µm for patient fibroblasts and 200 µm for progenitor fibroblasts. (**A5**) Time-course of collagen synthesis during vitamin C induction in fibroblast proliferation medium. (**B1**–**B4**) Endpoint imaging of Sirius Red staining for both primary fibroblast types following 7 days of vitamin C induction in keratinocyte proliferation medium. Scale bars = 400 µm for patient fibroblasts and 200 µm for progenitor fibroblasts. (**B5**) Time-course of collagen synthesis during vitamin C induction in keratinocyte proliferation medium. Results of the statistical analyses relative to collagen quantification experiments under vitamin C stimulation are presented in Table S2. CTRL, control group; Vit C, vitamin C.

For tangible consideration of an autologous to allogeneic substitution of the fibroblast cell source in CDEAs, in vitro benchmarking of critical functional attributes was necessary. Therefore, the potential of both primary fibroblast types for in vitro collagen synthesis under vitamin C induction was comparatively assessed in two types of culture media (i.e., fibroblast and keratinocyte proliferation medium, respectively). The results indicated that patient fibroblasts and FE002-SK2 primary progenitor fibroblasts were functionally stimulated (i.e., significantly increased collagen synthesis after 7 days) by the addition of vitamin C in all conditions (Figure 1, Table S2).

Overall, a trend of higher collagen synthesis was exhibited in the groups of primary cells which were induced in keratinocyte proliferation medium compared to the fibroblast proliferation medium (Figure 1B). Additionally, the differences in endpoint collagen levels between the cell types were less pronounced in the keratinocyte proliferation medium (Figure 1B). Furthermore, it was noted that the baseline collagen synthesis levels of the FE002-SK2 primary progenitor fibroblasts were systematically higher than those of the primary patient fibroblasts (i.e., all experimental groups and conditions, Figure 1). The results indicated that the FE002-SK2 primary progenitor fibroblasts synthesized more than twice the amount of collagen in 7 days of culture/induction in fibroblast medium compared to patient fibroblasts (Figure 1(A5)). The optimal technical specifications for dermal template manufacture (i.e., maximization of collagen synthesis) comprised vitamin C induction of FE002-SK2 primary progenitor fibroblasts for 7 days in keratinocyte proliferation medium (Figure 1). Therefore, these specifications were used for the manufacture of the functional allogeneic dermal template component in DE-FE002-SK2 constructs.

### 3.2. DE-FE002-SK2 Constructs Can Be Manufactured for Clinical Use in Three Weeks

In addition to collagen-producing functions, another main advantage of using an off-the-freezer allogeneic cell source resides in the temporal decoupling of lengthy cell manufacturing phases from the clinical workflow. Clinical grade cryopreserved FE002-SK2 cell stocks (e.g., for PBB batches) were used for a brief monolayer expansion phase before vitamin C stimulation for rapid allogeneic dermal template preparation [37]. With a targeted cellular expansion time of 7 days, various culture technical specifications were tested. The various culture groups were monitored by three experienced operators and were maintained until confluency levels of 95–100% were reached. The results indicated that a cell seeding density of 6000 cells/cm^2^ produced confluent monolayers after 7 days of culture (Table 1).

Notably, preliminary experiments had shown that a confluency level of 95–100% was optimal before the initiation of vitamin C induction in order to obtain maximized collagen synthesis. Therein, FE002-SK2 primary progenitor fibroblasts were found to spontaneously form multilayer sheets, which were assessed to optimally conform to the considered subsequent use as a dermal template. The obtention of 95–100% confluent cultures in 7 days was consistently achieved throughout passages 5–10 in the retained experimental setup and up to passage 12 in GMP manufacturing campaigns (i.e., end of production cell banks, EOPCB, Figure S4).

As previously reported, appropriate multi-tiered FE002 primary progenitor cell banking strategies enable the potential obtention of sufficient clinical grade materials to sustainably produce hundreds of millions of cell-based therapies, largely outweighing the number of patients that could practically be treated with such biologicals [39]. Within clinical investigation protocols for PBB lot preparation, FE002-SK2 progenitor fibroblasts are extemporaneously thawed to constitute the topical PBB constructs [37]. The cell-bearing constructs are made available after 18–24 h for clinical application over the first two weeks of patient treatment [37]. Therefore, additional FE002-SK2 primary progenitor fibroblasts may be simultaneously initiated from storage, in view of parallel DE-FE002-SK2 construct lot preparation for subsequent application.

**Figure 2:**
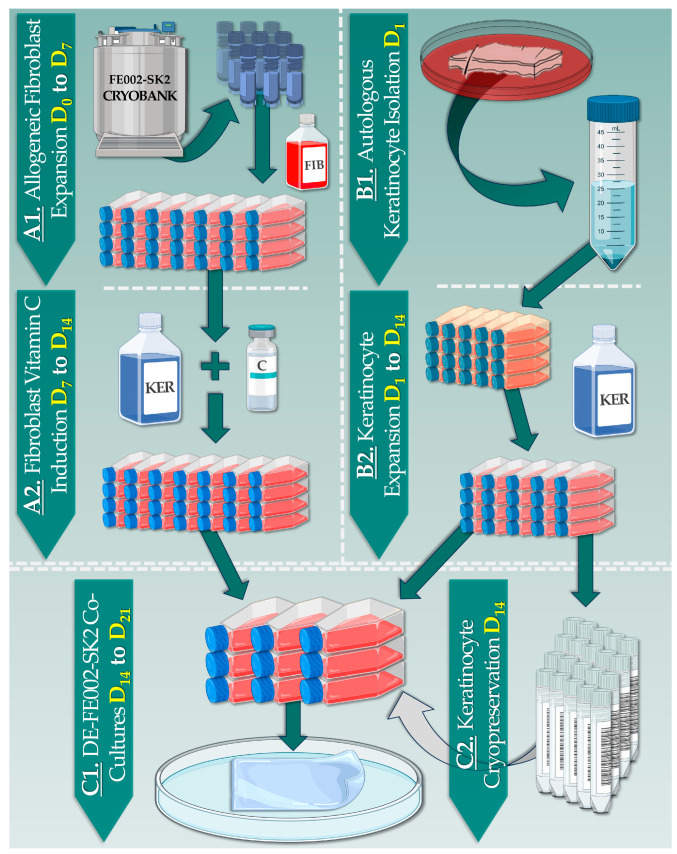
Schematic process detailing the parallel and sequential phases of DE-FE002-SK2 construct preparation. (**A1**) Using optimized technical specifications, allogeneic FE002-SK2 fibroblasts are expanded to confluency. (**A2**) For stimulation of collagen production, fibroblast cultures are treated with vitamin C. (**B1**) Upon epidermal biopsy reception, autologous keratinocyte isolation is rapidly performed. (**B2**) Autologous keratinocytes are cultured until the allogeneic dermal template is formed. (**C1**) The allogeneic dermal template and the autologous epidermal components are combined and further co-cultured in view of finished DE-FE002-SK2 construct formation. (**C2**) The excess autologous keratinocytes are cryopreserved and may be subsequently used to rapidly prepare new batches of DE-FE002-SK2 constructs. C, vitamin C; D, day; FIB, fibroblast proliferation medium; KER, keratinocyte proliferation medium.

The optimized FE002-SK2 primary progenitor fibroblast expansion data and the identified bottlenecks for CEA and CDEA production allowed for the establishment of a process for DE-FE002-SK2 construct preparation under GMP (Table 1, Figure 2).

Specifically, initiation of FE002-SK2 cells for allogeneic dermal template preparation may be performed at the same time as autologous epidermal biopsy harvesting (Figure 2). As no dermal biopsy harvesting is necessary within the novel combinational protocol (i.e., use of allogeneic fibroblasts), the patient donor site morbidity factor is reduced (Figure 2). Then, from a 4–10 cm^2^ epidermal biopsy, autologous keratinocytes may be isolated and expanded in vitro over a period of two weeks. This timeframe is sufficient for the parallel preparation of the allogeneic dermal template component (i.e., allogeneic fibroblast proliferation and functional induction, Figure 2(A1,A2)). Once both components have appropriately reached maturity, the expanded autologous keratinocytes are transferred via cell seeding to the top of the allogeneic dermal template and the co-cultures are maintained for one week before clinical delivery (Figure 2(C1)). In detail, DE-FE002-SK2 construct delivery can be arranged after 4–7 days of the final co-culture to allow for flexible organization of the reconstructive surgery. Overall and importantly, the optimized DE-FE002-SK2 manufacturing process enables clinical delivery within 16–21 days of autologous epidermal biopsy harvest, which constitutes a significant improvement over the 6–8-week delay of the standard CDEA manufacturing protocol (Figure 2).

### 3.3. DE-FE002-SK2 Constructs Display Structural Attributes Which Are Equivalent or Superior to Fully Autologous CDEAs

Parallel preparation of CDEAs using the standard protocol and of DE-FE002-SK2 constructs using the established protocol enabled us to technically benchmark both complex graft types using parametric gradings (Figure 2, Table 2).

**Figure 3:**
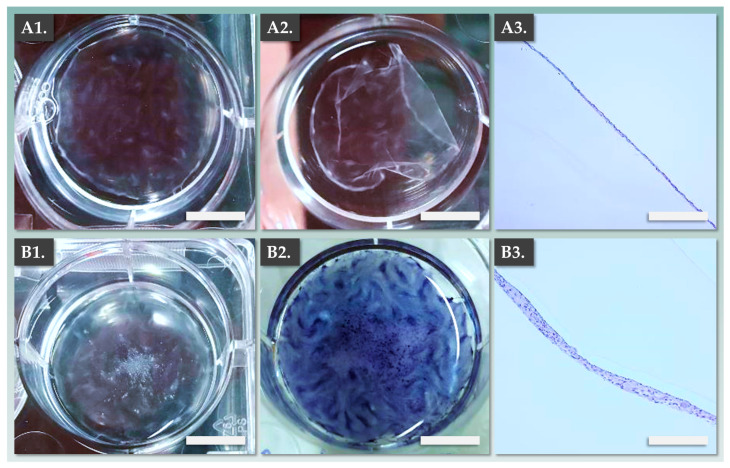
Macroscopic and microscopic records of allogeneic dermal templates and of the DE-FE002-SK2 constructs in formation. (**A1**) FE002-SK2-based dermal template following vitamin C induction. Scale bar = 5 mm. (**A2**) Detached FE002-SK2-based dermal template in PBS. Scale bar = 5 mm. (**A3**) Harris hematoxylin staining of the FE002-SK2-based dermal template. Scale bar = 300 µm. (**B1**) Fully formed FE002-SK2-based dermal template after 1 day of co-culture with patient primary keratinocytes. Scale bar = 5 mm. (**B2**) Combined DE-FE002-SK2 construct in co-culture following endpoint MTT staining. Scale bar = 5 mm. (**B3**) Harris hematoxylin staining of the fully formed combined DE-FE002-SK2 construct, showing stratified epidermal component formation. Scale bar = 300 µm. PBS, phosphate-buffered saline.

Illustrated records of these assessments are presented in Figure 3. In the allogeneic dermal template group, vitamin C-induced FE002-SK2 primary progenitor fibroblasts formed homogeneous multi-layers (Figure 3(A1)). The resulting sheets could then be detached and were processed for analysis, which confirmed the homogeneity of important structural attributes (Figure 3(A2,A3)).

Then, transfer of patient primary keratinocytes to the top of the formed dermal templates resulted in appropriate passive combination during the co-culture phase (Figure 3(B1–B3), Table 2). Importantly, it was confirmed that the epidermal component of the DE-FE002-SK2 constructs was composed of stratified keratinocytes (i.e., after 1 week of co-culture) and that the fully formed constructs could be detached and appropriately manipulated (Figure 3(B3)). Overall, the gathered experimental data enabled a thorough assessment of the DE-FE002-SK2 constructs, which display endpoint structural attributes which are equivalent or superior to autologous CDEA constructs (Table 2, Figure 3).

### 3.4. DE-FE002-SK2 Constructs Display Enhanced Bio-Adhesive Functions on DED Compared to CEAs

**Figure 4:**
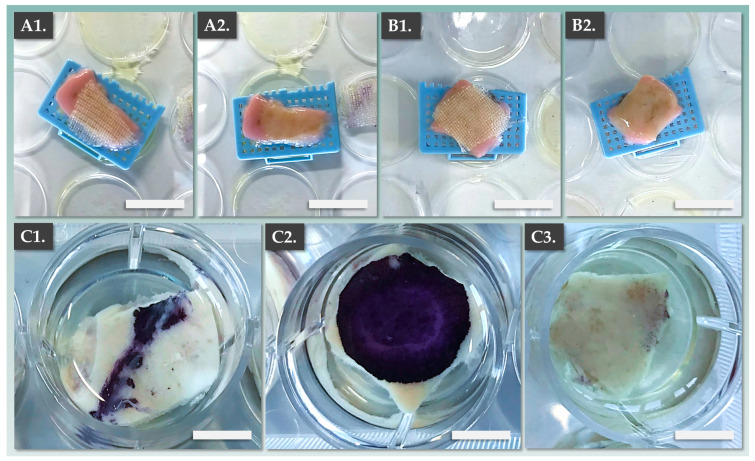
Assessment results of CEA and DE-FE002-SK2 construct adhesion capacities on a standardized ex vivo DED model. (**A1**,**A2**) Aspect of the DED model following topical CEA construct application and following gentle removal of the transport gauze. Scale bars = 10 mm. (**B1**,**B2**) Aspect of the DED model following topical DE-FE002-SK2 construct application and following gentle removal of the transport gauze. Scale bars = 10 mm. (**C1**) MTT staining of the model from the CEA group after 1 week of air–liquid organoculture, showing inhomogeneous graft take. Scale bar = 7 mm. (**C2**) MTT staining of the model from the DE-FE002-SK2 group after 1 week of air–liquid organoculture, showing homogeneous graft take. Scale bar = 7 mm. (**C3**) MTT staining of the model from the DED control group after 1 week of air–liquid organoculture. Scale bar = 7 mm. CEA, cultured epithelial autografts; DED, de-epidermalized dermis.

The important functional attributes of complex cell-based cutaneous grafts comprise the maintenance of structural integrity during handling and administration, as well as appropriate bio-adhesion and graft take. As DE-FE002-SK2 constructs could be rapidly obtained, several construct attributes of translational and functional importance were investigated further. Multiple CEA and DE-FE002-SK2 construct lots were prepared as described hereabove. The constructs were then transferred to a Vaseline gauze (i.e., transport scaffold) and were deposited on top of 1.0–2.5 cm^2^ DED models (Figure 4A,B). An illustrated stepwise overview of the preparation process for the DED model is presented in Figure S5.

Then, the transport gauze could be easily removed and constructs could be observed on top of the DED model (Figure 4A,B). Following topical graft application, the models were maintained in air–liquid organoculture for one week. Upon endpoint MTT staining, it was shown that the DE-FE002-SK2 constructs presented significantly enhanced bio-adhesion attributes compared to the standard CEAs (Figure 4C). Specifically, construct adhesion in the CEA group was found to be partial and inhomogeneous, with important CEA detachment at the end of the organoculture phase (Figure 4(C1)). Conversely, complete and homogeneous adhesion of the DE-FE002-SK2 preparations was observed (Figure 4(C2)). Such differential results may potentially be partly explained at a molecular level by the action (i.e., mediated by the presence of dermal fibroblasts) of basement membrane components (e.g., integrins, laminins, growth factors), which are known to possess important interface modulation functionalities [42]. Finally, comparative parametric assessments of CEA and DE-FE002-SK2 constructs were performed in terms of translational and functional attributes (Table 3).

**Figure 5:**
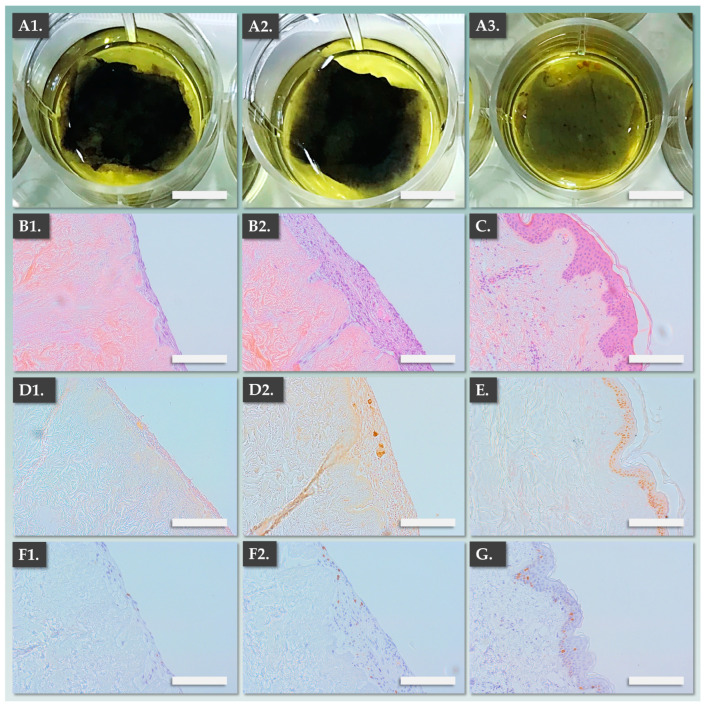
Functional investigation results for allogeneic dermal templates and fully formed DE-FE002-SK2 constructs on the ex vivo DED model. (**A1**–**A3**) MTT staining of the allogeneic dermal template on the DED, the DE-FE002-SK2 construct on the DED, and the DED control group following 1 week of air–liquid organoculture. Scale bars = 5 mm. (**B1**,**B2**) H&E staining of the allogeneic dermal template on the DED and the DE-FE002-SK2 construct on the DED. Scale bars = 150 µm. (**C**) H&E staining of the normal skin control group. Scale bar = 150 µm. (**D1**,**D2**) P63 staining of the allogeneic dermal template on the DED and the DE-FE002-SK2 construct on the DED. Scale bars = 150 µm. (**E**) P63 staining of the normal skin control group. Scale bar = 150 µm. (**F1**,**F2**) Ki67 staining of the allogeneic dermal template on the DED and the DE-FE002-SK2 construct on the DED. Scale bars = 150 µm. (**G**) Ki67 staining of the normal skin control group. DED, de-epidermalized dermis; H&E, hematoxylin and eosin.

For further mechanistic investigation into the bio-adhesive properties of the DE-FE002-SK2 constructs, the impact of autologous keratinocyte addition on the structural and functional attributes of the allogeneic dermal template was assessed using the DED model. Therefore, allogeneic dermal templates were prepared and divided into two groups. The first group consisted of the allogeneic dermal template alone (i.e., without keratinocytes) and the second group consisted of the fully formed DE-FE002-SK2 constructs. All samples were placed on fresh DED models and maintained in organoculture for one week. Endpoint MTT staining revealed that both experimental groups showed homogeneous adhesion on the DED model (Figure 5A).

Furthermore, and importantly, H&E staining of histological sections showed that both preparations presented multiple cell layers which were integrated within the DED (Figure 5(B1,B2)). Notably, it was not possible to obtain the corresponding data for CEA preparations, as these would not adhere to the DED model. Importantly, staining with the p63 marker (which specifically identifies keratinocytes) was observed in the upper to middle layers of the DE-FE002-SK2 preparations, confirming the bilayer structure of the complex grafts (Figure 5(D2)). Finally, the results showed that Ki67-positive cells (i.e., proliferating cells) were present in both graft components, confirming the maintenance of cellular functions following application on the DED (Figure 5(F1,F2)).

Overall, it was confirmed that the bio-adhesive functions of DE-FE002-SK2 constructs were conferred by the allogeneic dermal template, which enabled a significantly enhanced graft take (Figure 4 and Figure 5). Importantly, the results of ex vivo adhesion assays showed that allogeneic dermal templates based on functionally induced FE002-SK2 primary progenitor fibroblasts could potentially serve as standalone, rapidly obtainable under GMP, and universal dermal coverings for subsequent overlaying with a variety of epidermal substrates (Figure 5).

## 4. Discussion

### 4.1. Historical Evolution and Technical Bottlenecks in Burn Patient Cytotherapeutic Care

Notable advances in burn care and plastic surgery and specific improvements to techniques to assure tegument cover and quality have recently been emphasized [1,2,3,4,5,6]. Therein, a critical clinical objective is to cover the patient as rapidly as possible for optimized infection control. Secondly, esthetic and functional outcomes have become key components of modern rehabilitation pathways, as increasing numbers of severely burned patients return to regular activities [27].

From a skin transplant point-of-view, early allotransplantation began over 60 years ago, with hallmark advances made by Medawar in treating WWII burn victims [43]. He was able to present data on immune tolerance and transplantation, which provided the notion of immune privilege. He was awarded the Nobel Prize in 1960 for modern transplantation immunology and for providing the fundamental basis for showing that skin from one patient cannot be readily grafted to another patient [44]. Despite this described rejection process, allogeneic skin is still widely used as a functional biological dressing for temporary wound cover, assuring the necessary fluid balance and preparing the wound bed for subsequent autologous grafting and burn wound closure [17,18,19]. Conversely, allogeneic skin grafts are not advantageous for long-term cover due to secondary graft rejection, infection, and scar tissue formation [12,17,18].

Due to the lack of availability of human cadaver skin, development efforts have been directed toward temporary skin covers using artificial or xenogeneic products. Widely clinically applied porcine skin grafts (e.g., sterile and viable split-skin grafts from neonatal pigs) have been continuously developed as a source of high-quality and functional coverings for clinical use in human medicine [45,46]. More recently, natural sourcing of clinical grade biological coverage materials has been demonstrated from Nile Tilapia fish (i.e., *Orechromis niloticus*) skin. Similarly, North Atlantic cod (i.e., *Gadus morhua*) skin was first approved by the US FDA in 2013 (i.e., Kerecis Omega 3 dressings) despite the higher costs if used to treat high-TBSA burn patients [47,48,49]. From a cytotherapeutic viewpoint, several literature reviews have described the evolution of autologous keratinocyte culture use for severe burn wound management [1,2,3,4,21,22,23,24]. Despite the availability of formulation options (e.g., cell sheets and cell sprays), several weeks of culture remain necessary for therapeutic cell manufacture [24,27,28,29,30]. Notably, several clinical groups have studied the use of allogeneic keratinocytes (i.e., cultured and frozen) for initial treatments, yet these preparations tended to behave like cadaver skin and had to be replaced rapidly with autologous preparations [18].

Alternative projects have focused on genetically modified keratinocytes isolated from allogeneic sources [50]. Despite substantial progress in understanding immune competence and graft rejection, it is not yet possible to achieve immune compatibility [51]. Further understanding of immunological responses to modified allogeneic keratinocytes will help to develop potential clinical applications. Therefore, a variety of experimental methods are currently studied to obtain allogeneic keratinocytes which can be used universally and that may overcome host cellular immune responses [52]. However, depending on the experimental methods (e.g., use of viral vectors), other safety issues arise, such as the duration of transgene expression, vector immunogenicity, and vector tropism.

### 4.2. Transitioning to Complex Cutaneous Grafts for Enhanced Burn Victim Clinical Outcomes

Translational research groups have focused on artificial dermal matrices developed with collagens, polymers, and foams, which are adapted for association with therapeutic cell sources [2,4,6,7,8,10]. Therein, degradation of exogeneous substrates and parallel remodeling of autologous tissues are key factors. The overall performance in esthetics, functionality, and immune compatibility determines the level of success of allogeneic skin graft approaches [27,29,30,31]. Notably, there have been many efforts directed toward the development of dermal substitutes [2,11]. While several products are clinically investigated and marketed, there is no commercially available substrate which can sufficiently restore the functions of the skin. Specifically, an optimal substitute should ensure early restoration of anatomical and physiological skin functions, a feat which can currently only be provided by using a full-thickness skin graft or flap reconstruction protocols [11,12].

Therefore, strong emphasis has been placed on cultured cells to reconstitute an optimized tegument, with or without a dermal substrate or template [3,4,5,9,12,22]. From a functional and esthetic standpoint, it is generally accepted that including a dermal component (i.e., such as dermal fibroblasts) would significantly aid in graft take. This combinational approach has been studied using various allogeneic tissues and autologous cells, yet technical bottlenecks remain (i.e., differences in manufacturing timeframe requirements) [21,22,23,24,26,27,33]. Therefore, much room remains for further cutaneous cell therapy optimization, primarily to ensure full wound closure in a short time-period following injury to decrease the risk of infection.

As regards the use of allogeneic skin grafting materials in the Lausanne Burn Center, initial case studies date back to 1992 [17]. These cases were managed using the Cuono technique, with allogeneic cadaver skin bearing cultured autologous keratinocytes. The need for immunosuppressive therapy was evident due to the grafting of cadaver skin. Similarly, Damour et al. employed cultured allogeneic keratinocytes (i.e., the adapted Cuono technique) in 18 burn patients from 1998 to 2000 in France [18,19]. Namely, from one single cadaver, they were able to culture 30 m^2^ of epidermis [18]. Based on these advances, the standard CDEA protocols were developed and implemented in the CHUV in 1998, enabling the elimination of cadaver skin and the inclusion of functionally induced fibroblasts as dermal template components [20,27]. As the original protocols of Reinwald and Green for CEA preparation had already been implemented in the CHUV in 1985, early and rapid clinical adoption of novel topical cell therapies has constituted a landmark in Lausanne [13,14,15,16,17].

From a technical standpoint, a major drawback linked to the inclusion of autologous dermal fibroblasts in the CDEA manufacturing process is the lengthy manufacturing timeframe (i.e., practically doubled compared to CEAs). Of note, CEA manufacture was reported to require an average of 22.9 ± 4.2 days, while CDEA manufacture required an average of 50.0 ± 8.5 days in a patient cohort study from 2016 to 2018 (Table S1) [27]. Therefore, patients were transiently covered with Integra matrices before the CDEA cultures were ready for use. Additionally, clinically available cell therapy quantities are technically limited by the respective manufacturing lot sizes. Therein, a maximum of 0.4 m^2^ of skin grafts may be manufactured at one given time (i.e., approximately 50 units of T75 culture flasks for CEAs and 40 units of T75 culture flasks for CDEAs, Table S1) [27]. Therefore, alternative and readily available (e.g., off-the-freezer) therapeutic cell sources would be of particular importance in the early management of severe burn trauma and could potentially help save lives.

### 4.3. Dermal Components Are Necessary for High-Quality Closure of Extensive and Deep Burns

Skin grafting is mandatory in extensive, deep second and third degree burns because the endogenous cellular components necessary for cutaneous regeneration are damaged. Extensive burns generate a disturbance of tissue oxygenation as well as fluid and protein losses, which increase the risk of dehydration and infections. This is why it is important to rapidly cover the wounds to support their repair and limit the related consequences. We have previously reported the surgical management of two seriously burned patients (i.e., 92% and 90% TBSA, respectively) with a 17-year interval [27]. Differences in treatment regimens led to a significant difference in hospitalization stay. Therein, the first patient benefited from both CEA and CDEA preparations at various stages of surgical reconstruction [27]. The second patient was able to benefit from early allogeneic cellular therapies with the use of PBBs to replace cadaver skin and to prepare the wound beds [27].

The second patient was subsequently treated with CEA preparations only (i.e., no CDEAs), as his hospital stay in the ICU was reduced to 76 days compared to 162 days for the first patient [27]. Standardized monitoring of skin reconstruction quality revealed that the use of CDEAs resulted in a markedly superior cosmetic appearance and a significantly higher elastic recovery as compared to CEA use only (Figure S6). These findings indicated that by reconstructing dermal and epidermal structures, both esthetic and significant functional improvements could additionally be obtained over time. These in-house clinical observations were in line with those of Lamme et al., who noted that the improvement in wound healing was correlated with higher numbers of fibroblasts within the applied dermal substitute [34]. Therefore, the use of appropriate dermal components in complex wound care may demonstrably enhance quality-related clinical outcomes.

### 4.4. Allogeneic FE002-SK2 Primary Progenitor Fibroblasts Have Been Extensively Clinically Tested

Primary progenitor cells are differentiated cells, notably differing from stem cells in terms of differentiation potential [35]. They are highly resistant to oxidative stress and have minimal nutritional requirements, while having high proliferation potentials and low immunogenic properties [39]. Formulated in temporary topical PBB constructs, such cells are thought to mainly act by means of tissue repair/regeneration mediation [35,37,39]. The absence of therapeutic cell engraftment was evidenced in early clinical studies, where biopsies were harvested from a female patient having received multiple male progenitor cell topical applications [35]. Such elements were considered positively from a safety viewpoint, in conjunction with the fact that no clinical evidence of immunological rejection of primary FE002 cells was reported to date [37,53]. Specifically, the immune privilege of FE002 primary progenitor cells and of similar cell sources was studied in vitro (e.g., optimal HLA marker panels) and using various immunological settings in vivo (e.g., use of FE002 primary chondroprogenitors in caprine and mice models) [35,39,54,55]. However, while the considered DE-FE002-SK2 constructs (i.e., tissue engineering products) are designed to engraft in the host, further long-term in vivo studies would be warranted to optimally document safety attributes, building on the existing body of knowledge around FE002 allogeneic biologicals [53].

The main therapeutic objective of using PBBs is to stimulate the natural cutaneous healing process of treated burn wounds during the first ten to twelve days, and thus potentially avoid the need for an autograft or at least reduce the size of the grafted area. Subsequently, in cases where skin autografts are required, PBBs may be further applied for promoting re-epithelialization of donor site wounds. It was shown that the early use of PBBs resulted in the promotion of a qualitatively enhanced cutaneous healing process, with drastically improved esthetic and functional outcomes, and reduced the need for subsequent corrective interventions [4,5,37]. From a therapeutic viewpoint, PBBs were developed and have been clinically applied in Lausanne since 2000–2005 [37]. Therein, no safety-related concerns have been raised and an enhanced repair tissue quality was evidenced [37]. From a regulatory standpoint, the use of FE002-SK2-based therapeutic materials in clinical investigation settings has thus far been approved by the US FDA, the Taiwanese FDA, the Japanese PMDA, and Swissmedic [39].

Based on the available scientific, technical, and clinical hindsight on the clinical grade FE002-SK2 primary progenitor cell source, its inclusion as a dermal component of a novel combinational cutaneous graft (e.g., DE-FE002-SK2 construct) may tangibly be considered [35,36,37,38,39]. Notably, in vitro functional induction of dermal fibroblast sources has been reported since the early 1970s, when it was shown that vitamin C could enhance collagen synthesis [40]. Individual experiments comparing adult fibroblasts to FE002-SK2 primary progenitor cells showed that the FE002 progenitor cells possess significant innate collagen synthesis capacities (Figure 1). Such attributes were further studied, harnessed, and potentiated in the present study for the obtention of standardized and optimized functionality within allogeneic dermal template manufacturing (Figure 2).

### 4.5. DE-FE002-SK2 Constructs: Technically Enhanced Modern Alternatives to Standard CDEA Protocols

Vitamin C is known to functionally enhance extracellular matrix formation in vitro, whereas the FE002-SK2 cells constitutively possessed higher collagen production and deposition activities compared to patient fibroblasts (Figure 1). Stimulation with vitamin C potentiated extracellular matrix formation by the allogeneic cells, and enhanced bio-adhesion attributes of the DE-FE002-SK2 constructs were recorded (Figure 1, Figure 4 and Figure 5). Notably, the ex vivo DED model suggested an enhanced DE-FE002-SK2 graft take over CEAs, which have been used locally and internationally (supplied by the Lausanne Burn Center, e.g., Zurich-based clinical centers, UK, or Finland) as a standard of care. From a technical standpoint, while the retained MTT readout provided only basic information about the mitochondrial activity of the cells, the simplicity of the assay and the visual assessment of cellular presence and cellular homogeneity were assessed to be appropriate within the scope of the present proof-of-concept work (i.e., functionality optimization at the construct level).

Multiparametric advantages were set forth herein around the use of FE002-SK2 cells over autologous fibroblasts (i.e., sustainable off-the-freezer cell source, rapid production, constitutive collagen synthesis, and enhanced bio-adhesiveness). Comparative parametric assessments of CEAs or CDEAs and DE-FE002-SK2 constructs were performed in terms of translational and functional attributes (Table 2 and Table 3). Importantly, the structural integrity maintenance of the DE-FE002-SK2 constructs during standard handling (i.e., manufacturing- and clinical-application-related handling) was confirmed. Specifically, matured constructs (i.e., about 10–50 cm^2^) must be able to resist harvest, physical transfer and stapling to the Vaseline gauze, and transport to the clinic. Therefore, the DE-FE002-SK2 preparations displayed superiority in structural integrity retention over the CEA preparations, most likely partially due to the additional thickness/sturdiness of the complex cell sheet. Finally, as concerns the treatment of large TBSA burns, multiple DE-FE002-SK2 grafts would need to be serially manufactured and parallelly clinically applied, similarly to existing CDEA protocols. Therein, while the proposed bi-component cell-based constructs may even become available too early in the clinical pathway of severe burn patients (i.e., depending on the resuscitation and stabilization phases, with surgical wound bed preparation), their early use as temporary coverages and wound healing enhancers (i.e., first weeks of treatment) may potentially be considered. By extension, the proposed DE-FE002-SK2 constructs may also potentially be used in alternative complex and moderate cases of cutaneous affections (e.g., chronic lower limb ulcers) requiring high-quality repair interventions, taking as an example the clinical diversification already implemented in Lausanne for PBB constructs [53].

Overall, the original data reported herein have confirmed that the proposed use of allogeneic dermal progenitor fibroblasts within complex cutaneous graft manufacture addresses two critical issues of clinical relevance. Namely, the rapidity of wound coverage may be enhanced (i.e., off-the-freezer cell stock) and the structural/functional quality of cutaneous tissular repair may potentially be further optimized (i.e., early use of a dermal component and enhanced graft take). While further studies and in vivo investigative work are warranted for the proposed DE-FE002-SK2 constructs, transposition to GMP manufacturing settings and large-scale clinical translation are inherently confirmed by the locally acquired experience of the authors in cutaneous cytotherapies (i.e., CEAs, CDEAs, and PBBs). Such considerations further strengthen the rationale for working towards autologous to allogeneic clinical protocol transpositions, as the potential technical and clinical benefits are significant and quantifiable.

### 4.6. Study Limitations and Future Perspectives

Differential cell-based management approaches depend on the extent, depth, and related complications of the burn wounds. Overall, novel solutions may contribute to accelerating the recovery of burn wounds and to shortening wound healing processes, ideally with an improved quality of cutaneous scarring. Despite the technical and functional advantages of the DE-FE002-SK2 constructs, the latter remain limited by the simplicity of their cellular constituents and therefore do not optimally mimic healthy human skin. Further developments of grafts including more complex skin structures such as melanocytes, sweat glands, hair follicles, blood vessels, and lymphatic capillaries are needed, yet current approaches in this domain still lack robust clinical data [56,57,58,59]. From a transpositional viewpoint, such biomimicking applications would also require up-scaling, which will in all probability be limited by available manufacturing capabilities. Furthermore, an optimized scaffold or matrix for simplified graft preparation and delivery would be of high interest (i.e., in replacement of the Vaseline gauze). Appropriate scaffolds could potentially include thermolabile hydrogels for handling of the cell sheet or a biodegradable scaffold which could easily be removed during bandage exchanges.

From a methodological standpoint, the use of simple readouts in this study and related descriptive analyses did not enable us to characterize the mechanisms of action or the internal functional attributes of the proposed DE-FE002-SK2 constructs. Specifically, the scope of the present proof-of-concept work was built around function-oriented manufacturing process optimization, with a focus on manufacturing timeframe rationalization. Therefore, further investigation of the proposed DE-FE002-SK2 constructs at fundamental and mechanistic levels (i.e., using complementary biochemical, genomic, and proteomic methods) will allow for the optimal characterization of the proposed constructs and a tentative explanation of the mechanisms of action [60]. While the critical functional attributes of the complex grafts were confirmed (e.g., bio-adhesion capacities) in comparison to CEA or CDEA elements, the use of alternative methods for cellular activity determination (i.e., to complement the basic MTT readout) will potentially yield an enhanced understanding of key and critical functional attributes to eventually augment the quality of clinical outcomes. Therein, in addition to primary efficacy outcomes (e.g., time to complete wound closure), specific secondary outcomes related to the quality of tissular repair (e.g., biomechanical analysis of repaired skin and re-operation rates) are of prime interest for pertinent tissue engineering product efficacy benchmarking.

## 5. Conclusions

The aim of this study was to establish proofs-of-concept for a novel combinational topical tissue engineering product in view of clinical cutaneous cytotherapy workflow rationalization. Extensive local technical and clinical hindsight on the use of autologous and allogeneic skin cell sources for burn wound management was harnessed for the new manufacturing process design. It was first demonstrated that allogeneic FE002-SK2 dermal progenitor fibroblasts could be used instead of autologous fibroblasts for functional dermal template preparation, starting from a standardized cryopreserved source. Then, it was shown that DE-FE002-SK2 constructs could be manufactured in a condensed timeframe of 3 weeks, constituting a major technical advantage over the current 6–8-week-long fully autologous protocol. Ex vivo, the DE-FE002-SK2 constructs were found to be structurally equivalent to standard CDEAs and possessed enhanced bio-adhesion attributes compared to standard CEAs. Generally, the presented work constitutes a technical basis for further GMP transposition of the combinational DE-FE002-SK2 construct in view of investigative clinical work. Importantly, tangible consideration of the switch toward allogeneic cell sources for dermo-epidermal graft manufacture has been enabled by the landmark use of dermal progenitor fibroblasts (e.g., FE002-SK2 cell source) for burn wound care over the past decades in Lausanne. Overall, the rationalized manufacturing timeframes presented herein bear the potential to significantly optimize severe burn patient cytotherapeutic management workflows, potentially enabling faster wound closure and enhanced tissular healing quality.

## Figures and Tables

**Table 1:** Quantitative results of the optimization study for the allogeneic FE002-SK2 primary progenitor fibroblast expansion phase. For the needs of the experiments, cryopreserved FE002-SK2 primary progenitor fibroblasts at passage level 10 were used as cell seeding materials.

Parameters	Values/Results			
Cell seeding density (viable cells/cm^2^)	1500	3000	6000	20,000
Time to confluency (days)	12 ± 2	10 ± 2	7 ± 1	4 ± 0.5
Medium exchange procedures (n)	5	4	3	2
Seeding lot size ^1^ (10^6^ cells)	2.8	5.6	11.3	37.5

^1^ Calculated for 25 units of T75 cell culture flasks.

**Table 2:** Comparative assessment of the attributes of autologous CDEAs and of autologous/allogeneic DE-FE002-SK2 constructs. Assessments were recorded as mean multi-operator gradings at the end of the dermal template formation phase and at the end of the combined construct formation phase. CDEA, cultured dermo-epidermal autograft.

Process Phase	Parameters	Targets	Methods	Gradings ^1^	
				CDEA Group	DE-FE002-SK2 Group
1. Dermal Template	Manufacturing timeframe	Two weeks of culture	Operator assessment	−	+++
	Fibroblast monolayer formation after expansion	Formation of a homogeneous cellular layer	Operator assessment; microscopy	++	+++
	Dermal template sheet formation after vitamin C induction	Formation of a dermal template by collagen synthesis	Operator assessment; collagen staining	++	+++
	Homogeneity of the dermal template sheet	Consistency of dermal template attributes over the whole surface	Operator assessment; histology	++	+++
	Robustness of the dermal template sheet	Possibility to detach and manipulate the dermal template	Operator assessment; handling	+	+++
2. Combined Construct	Manufacturing timeframe	One week of culture	Operator assessment	+	+++
	Compatibility between the dermal template and autologous keratinocytes	Passive combination of components in co-culture	Operator assessment; histology	++	+++
	Stratification of the epidermal component	Formation of a stratified epidermal component	Operator assessment; histology	+	+++
	Homogeneity of the construct	Consistency of construct attributes over the whole surface	Operator assessment; histology	+	++
	Robustness of the construct	Possibility to detach and manipulate the construct	Operator assessment; handling	+	++

^1^ Gradings were attributed as follows: (+++) = conforming, excellent performance; (++) = conforming, good performance; (+) = conforming; (−) = non-conforming.

**Table 3:** Comparative assessment of translational and functional attributes of CEAs and DE-FE002-SK2 constructs within the ex vivo DED model. Assessments were recorded as mean multi-operator gradings at the time of construct topical application on the DED model and at the end of the organoculture phase. CEA, cultured epithelial autograft; DED, de-epidermalized dermis.

Attribute Type	Parameters	Targets	Methods	Gradings ^1^	
				CEA Group	DE-FE002-SK2 Group
1. Translational Attributes	Transfer of the construct to a Vaseline gauze	Possibility to manipulate and transport the construct using the gauze	Operator assessment	++	+++
	Application of the construct on the DED model	Simple topical application on the DED surface	Operator assessment	+++	+++
	Applied construct structural integrity maintenance	Maintenance of construct structural integrity on the DED model	Operator assessment	++	++
2. Functional Attributes	Initial adhesion of the construct to the DED model	Construct adheres rapidly to the DED model	Operator assessment	++	++
	Endpoint homogeneous adhesion of the construct to the DED model	Construct adheres homogeneously to the DED model	Operator assessment; MTT	−	++
	Endpoint homogeneous metabolic activity throughout of the construct on the DED model	Cellular metabolic activity is significant and homogeneous throughout the construct on the DED model	Operator assessment; MTT	±	+++

^1^ Gradings were attributed as follows: (+++) = conforming, excellent performance; (++) = conforming, good performance; (±) = unclear, additional data required; (−) = non-conforming.

## Data Availability

The data presented in this study are available upon written and reasonable request from the corresponding authors.

## Supplementary Materials

The following supporting information can be downloaded at: https://www.mdpi.com/article/10.3390/pharmaceutics15092334/s1, Figure S1: Clinical flowchart for burn patient management phases and treatments; Figure S2: Technical manufacturing workflow for CEA constructs; Figure S3: Technical manufacturing workflow for CDEA constructs; Figure S4: Technical workflow for multi-tiered primary progenitor fibroblast cell banking and PBB clinical lot preparation for use in the Lausanne Burn Center; Figure S5: Illustrated process for the ex vivo DED model preparation; Figure S6: Illustration for clinical use of CEA and CDEA constructs, with qualitative endpoints; Table S1: Comparative parameters and timeframes for manufacture of clinical batches of PBB, CEA, and CDEA constructs in the Lausanne University Hospital; Table S2: Results of statistical analyses for collagen quantification assays under vitamin C stimulation.

## Abbreviations

CDEA	cultured dermo-epidermal autograft
CEA	cultured epithelial autograft
CHUV	Centre Hospitalier Universitaire Vaudois
DED	de-epidermalized dermis
DE-FE002-SK2	bioengineered skin graft based on cultured allogeneic fibroblasts and autologous keratinocytes
DMEM	Dulbecco’s modified Eagle medium
DMSO	dimethyl sulfoxide
ECM	extracellular matrix
EDTA	ethylenediaminetetraacetic acid
EOPCB	end of production cell bank
FBS	fetal bovine serum
FE002-SK2	clinical grade primary progenitor fibroblast cell source
GMP	good manufacturing practices
H&E	hematoxylin and eosin
MCB	master cell bank
PBB	progenitor biological bandages
PBS	phosphate-buffered saline
PCB	parental cell bank
TBSA	total body surface area
UK	United Kingdom
USA	United States of America
WCB	working cell bank
