# Supplementary material for: Cutaneous Cell Therapy Manufacturing Timeframe Rationalization: Allogeneic Off-the-Freezer Fibroblasts for Dermo-Epidermal Combined Preparations (DE-FE002-SK2) in Burn Care
Source: Pharmaceutics. 2023 Sep 16;15(9):2334. doi: 10.3390/pharmaceutics15092334 (PMC10536166; doi:10.3390/pharmaceutics15092334)
Supplement: Supplementary file 1 [file pharmaceutics-15-02334-s001.zip › pharmaceutics-2586162-supplementary.pdf]

pharmaceutics

Supplementary Materials:

Cutaneous Cell Therapy Manufacturing Timeframe Rationalization: Allogeneic Off-the-Freezer Fibroblasts for Dermo-Epidermal Combined Preparations (DE-FE002-SK2) in Burn Care

Xi Chen, Alexis Laurent, Zhifeng Liao, Sandra Jaccoud, Philippe Abdel-Sayed, Marjorie Flahaut, Corinne Scaletta, Wassim Raffoul, Lee Ann Applegate and Nathalie Hirt-Burri

1. Supplementary Figures

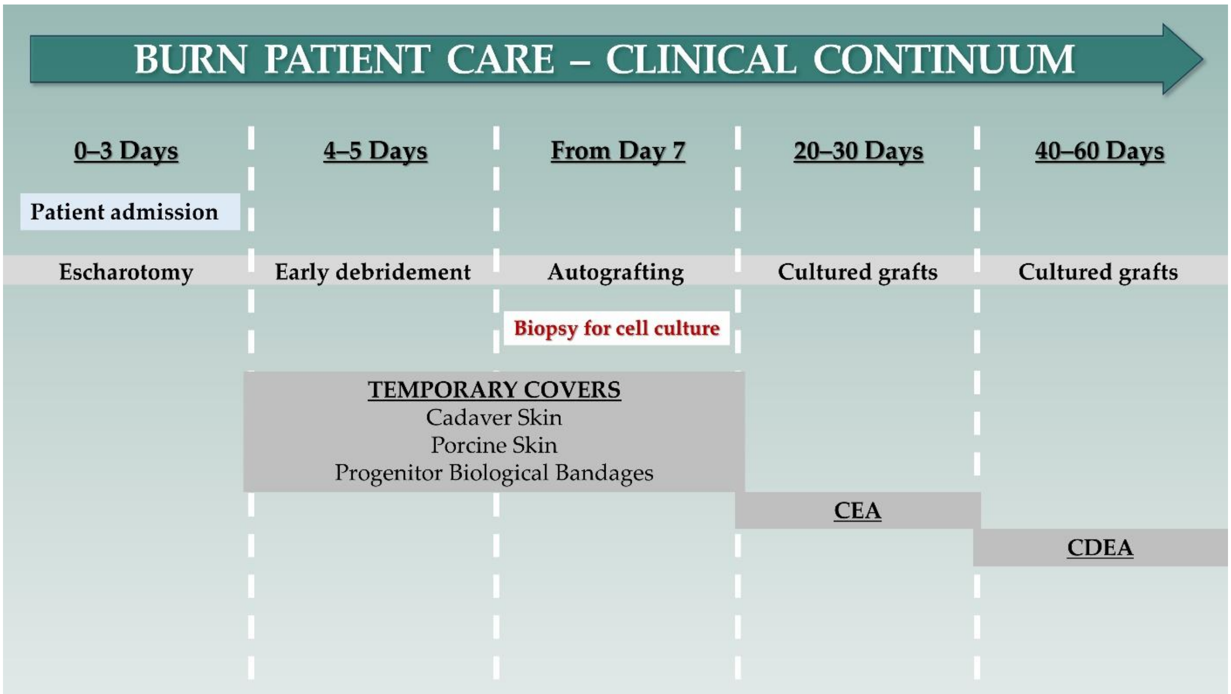

Figure S1. Flowchart illustrating the integrated clinical pathway for severely burned patients in the Lausanne University Hospital Burn Center (Lausanne, Switzerland). Specifically, the current sequential and integrated phases of burn wound surgical management and cutaneous cell therapy (e.g., CEA, CDEA, PBB) administration are presented. CEA, cultured epithelial autografts; CDEA, cultured dermo-epidermal autografts; PBB, progenitor biological bandages.

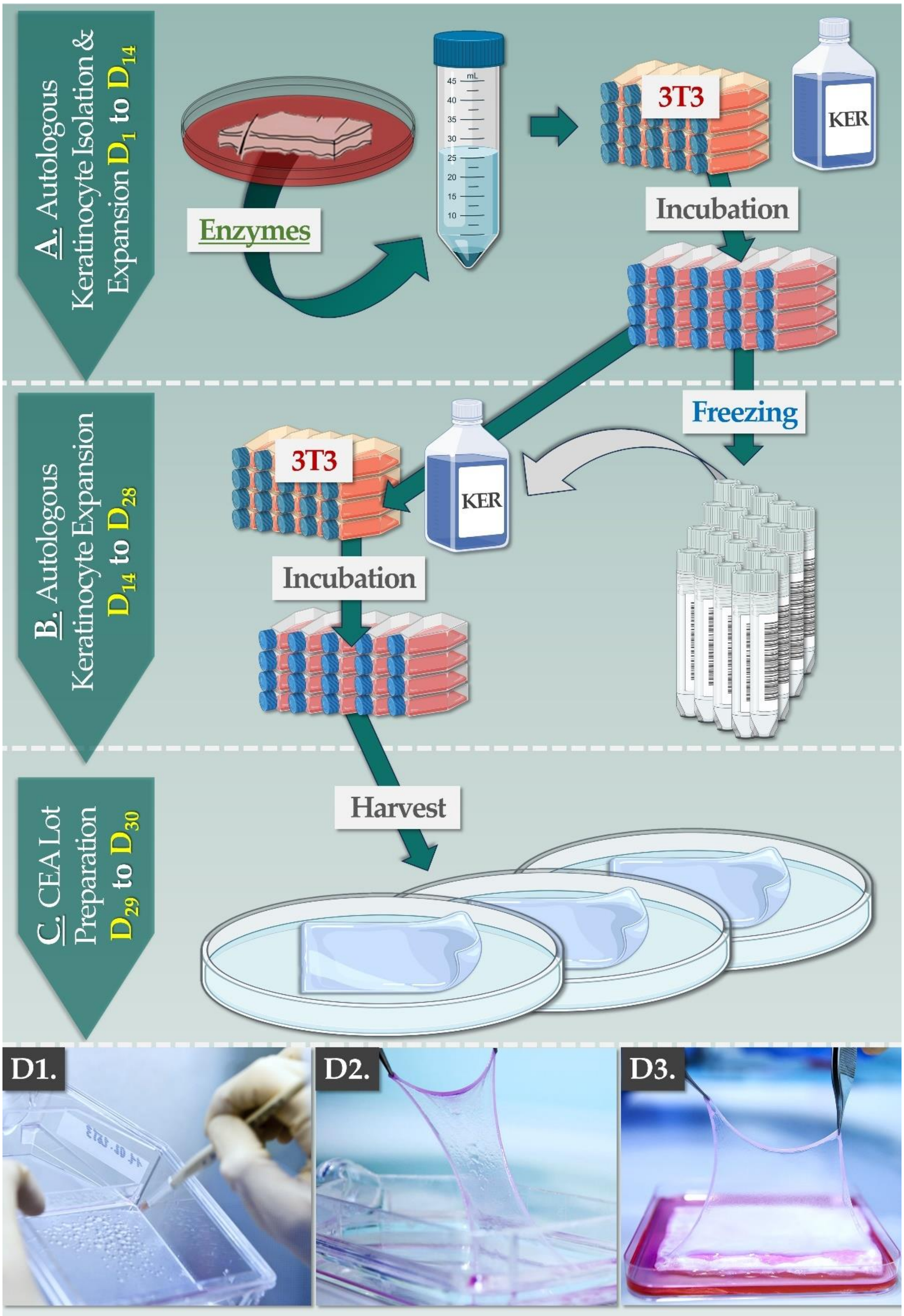

Figure S2. Illustration of the standard CEA manufacturing process as implemented in the Lausanne University Hospital since 1985. (**A**) From an autologous epidermal biopsy of 4–10 cm^2, autologous keratinocytes are enzymatically isolated and expanded in vitro. (**B**) The obtained autologous keratinocytes are either re-expanded in vitro or cryopreserved for further autograft manufacturing activities. Notably, subsequent CEA clinical lots may be generated from cryopreserved autologous keratinocyte stocks, without the need for repeat biopsy harvest. (**C**) The stratified autologous keratinocyte sheets are harvested and appropriately formulated for topical clinical delivery on patient cutaneous wounds. (**D1**) Manual detachment of stratified keratinocyte cultures. (**D2**) Harvest and transfer of stratified keratinocyte cultures. (**D3**) Mounting of stratified keratinocyte cultures on the transport Vaseline gauze. CEA, cultured epithelial autografts; KER, keratinocyte proliferation medium.

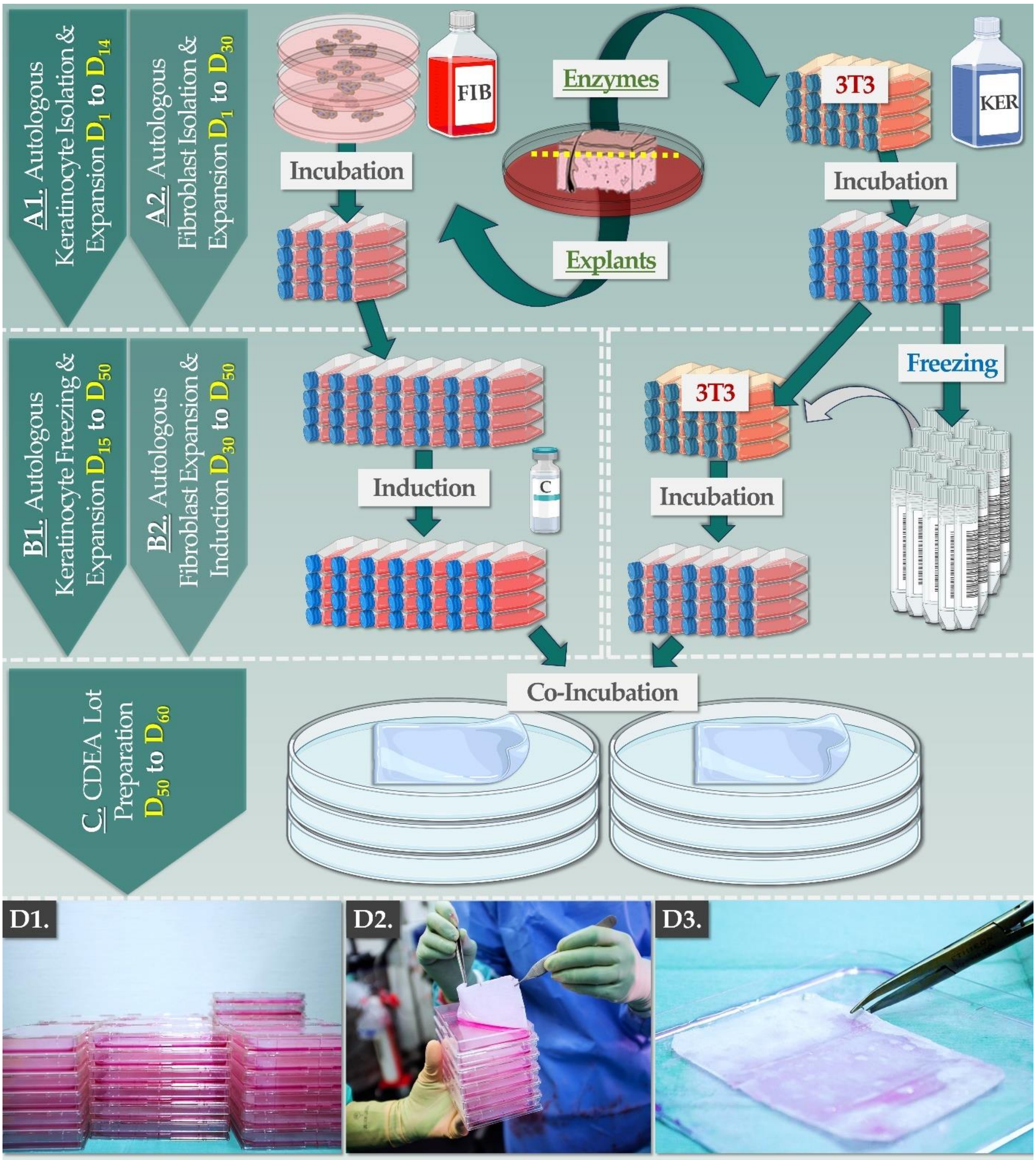

Figure S3. Illustration of the standard CDEA manufacturing process as implemented in the Lausanne University Hospital since 1998. (**A1**) Autologous keratinocytes are isolated and cultured in the same way as for CEAs, using the epidermal portion of the skin biopsy. (**A2**) Autologous fibroblasts are isolated from the dermal portion of the skin biopsy and are cultured using an explant method. (**B1**) Autologous keratinocytes are serially expanded and cryopreserved as required to generate sufficient amounts for the clinical CDEA construct lot. (**B2**) Autologous fibroblasts are expanded as required to generate sufficient amounts for the clinical CDEA construct lot and are finally functionally induced for 7–10 days with vitamin C. (**C**) Close coordination of the various culture types enables to simultaneously harvest mature autologous dermal templates and autologous keratinocytes for the last manufacturing phase. Following 7–10 days of co-culture, the bi-layer CDEA constructs are harvested and are appropriately formulated for topical clinical delivery on patient cutaneous wounds. (**D1**) Clinical lot of autologous cutaneous constructs. (**D2**) Construct lot handling in the clinic. (**D3**) Individual cutaneous construct handling in the clinic. C, vitamin C; CDEA, cultured dermo-epidermal autografts; CEA, cultured epithelial autografts; FIB, fibroblast proliferation medium; KER, keratinocyte proliferation medium.

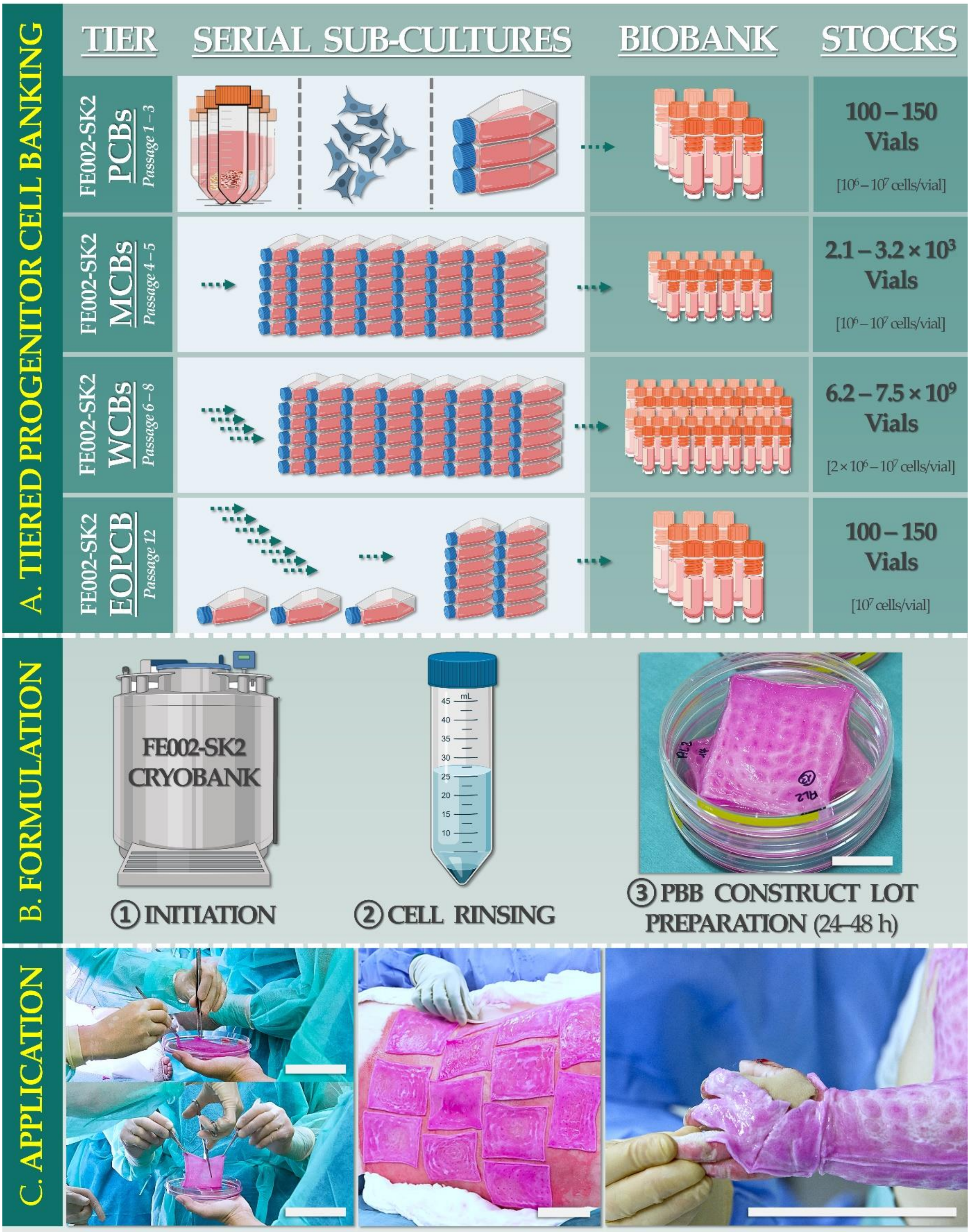

Figure S4. Technical workflow for multi-tiered primary progenitor fibroblast cell banking (e.g., clinical grade FE002-SK2 cell source) and PBB clinical lot preparation for therapeutic use in the Lausanne Burn Center. (**A**) Multi-tiered primary progenitor cell banking enables to sustainably generate vast quantities of homogeneous and consistent clinical grade therapeutic cellular materials. Importantly, consistent clinical grade cell lots (i.e., same passage number) are used for GMP cytotherapeutic PBB construct manufacture. (**B**) Following off-the-freezer initiation, the therapeutic FE002-SK2 cells are rinsed, dispensed on equine collagen sheet scaffolds to form PBB constructs, and allowed to recover for 24–48 h in a controlled atmosphere at 37 °C. Scale bar = 4 cm. (**C**) PBB constructs are formatted as required by the surgical team and are applied over the burn wound or skin donor-site wound surface. Scale bars = 10 cm. EOPCB, end of production cell bank; GMP, good manufacturing practices; h, hours; MCB, master cell bank; PBB, progenitor biological bandages; PCB, parental cell bank; WCB, working cell bank.

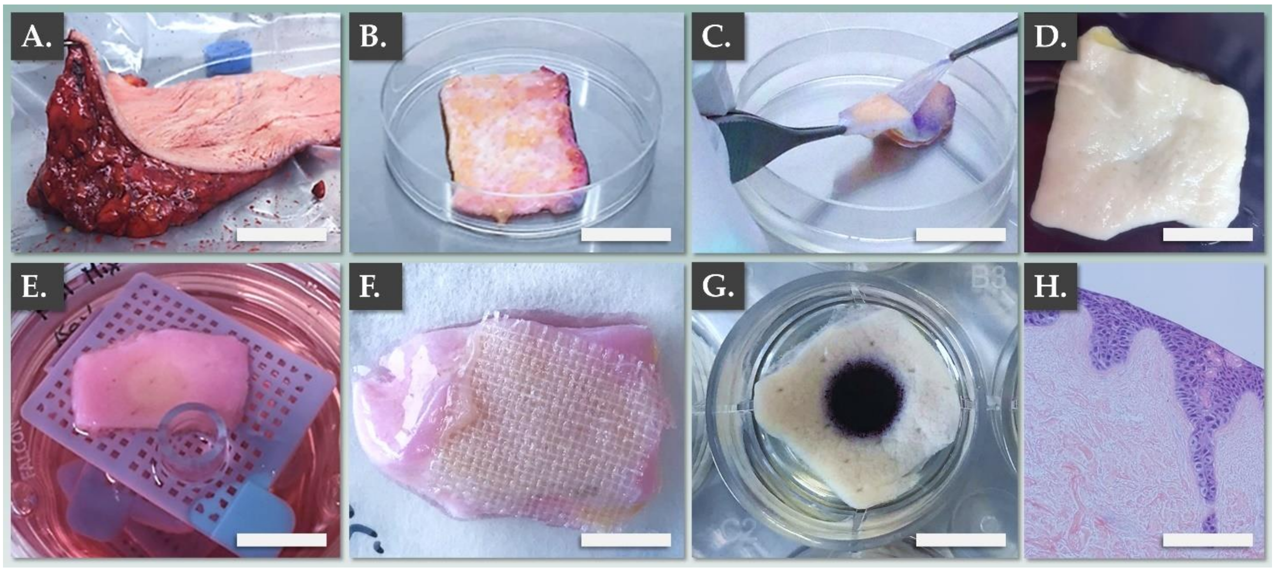

Figure S5. Illustrated workflow for the ex vivo DED model preparation. (**A**) The underlaying fat layer (e.g., in abdominoplasty skin biopsies) is firstly removed using a scalpel. Scale bar = 4 cm. (**B**) Small residual fat fragments are removed from the prepared skin portion. Scale bar = 4 cm. (**C**) The prepared skin is dissected into 1–2 cm^2 sections, which are incubated for at least 24 h (i.e., generally sufficient) and up to 72 h (i.e., depending on the sample) in 1 M NaCl at 37 °C. The epidermis is then delicately removed from the dermis using forceps. Scale bar = 4 cm. (**D**) The resulting DED units are washed several times with 1× PBS and are stored at 4 °C until use. It was validated that DED units may be used for up to one year following preparation and conservation at 4 °C. Scale bar = 5 mm. (**E**) For the experiments, the DED units are transferred into 12-well plates with 1 mL of keratinocyte proliferation medium/well and are incubated at 37 °C under 5% CO_2 for 24 h. Then, cell-based constructs or cell suspensions (e.g., FE002-SK2 primary progenitor fibroblasts or primary keratinocytes, 100 μL) are deposited on the DED. Cell suspensions are dispensed in an 8-mm diameter glass insert. Following 3 days of incubation to enable optimal cell attachment, the glass inserts are removed. The DED units bearing the samples are transferred on a plastic grid, to form an air-liquid interface. Keratinocyte proliferation medium is placed in the well and is level with the top of the grid. The medium is exchanged twice weekly. Scale bar = 10 mm. (**F**) For fully formed constructs, sample application on the DED model is performed using the transport Vaseline gauze, which is then gently removed. Scale bar = 5 mm. (**G**) Endpoint MTT assessments may be performed to confirm cellular metabolic activity maintenance and colonization or bio-adhesion homogeneity. Scale bar = 8 mm. (**H**) Endpoint histological assessments may be performed to investigate structural and bio-integration attributes. Scale bar = 200 μm. DED, de-epidermalized dermis; PBS, phosphate-buffered saline.

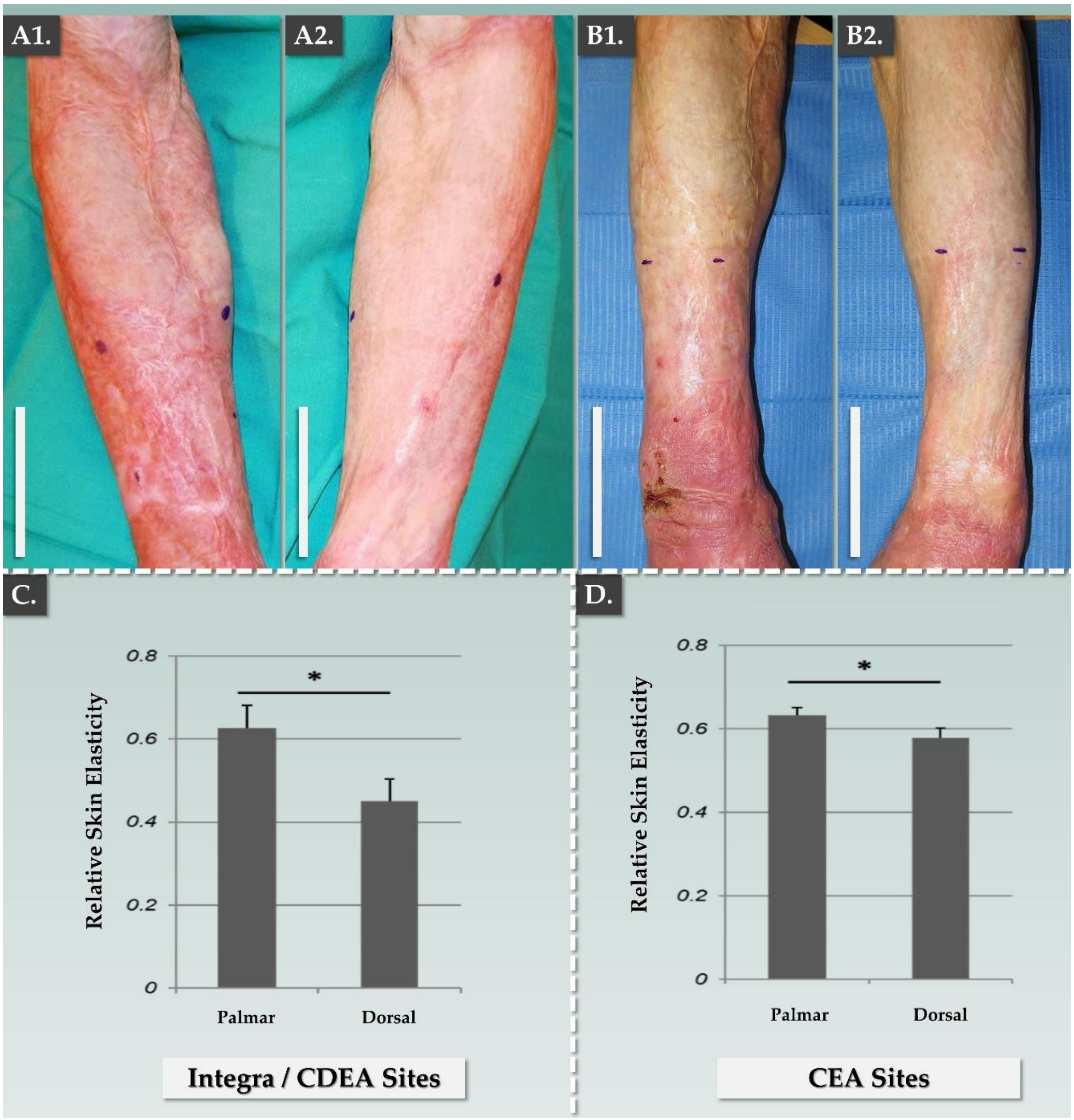

Figure S6. Illustration for the clinical use of CEA and CDEA constructs, with qualitative endpoints. The 22-year-old male patient (i.e., 92% TBSA) presented severe 3rd degree burns on both arms. The patient received either CEAs (i.e., right arm) or Integra followed by CDEAs (i.e., left arm) for skin reconstruction. (**A1**,**A2**) Follow-up pictures after initial wound closure. Scale bars = 5 cm. (**B1**,**B2**) Follow-up pictures after a time-period of 16 years post-wound closure. Scale bars = 5 cm. Overall, skin quality and pigmentation were better following CDEA treatment, based on clinical follow-up records. Namely, CDEA treatment resulted in markedly superior cosmetic appearance with enhanced biophysical properties (i.e., comparative viscoelasticity measurement data). In detail, the biomechanical status of the skin was quantified (i.e., Cutometer MPA580 system) ten years after reconstruction. The Cutometer system was used to apply 500 mbar of negative pressure through a 6-mm diameter aperture on various skin sites (i.e., palmar and dorsal aspects of the forearms) for three seconds, followed by three seconds at ambient pressure, for four consecutive cycles on each test site. (**C**) Relative elastic recovery of skin tissue reconstructed with Integra + CDEAs. The difference in mean values was statistically significant (i.e., "*", $p < 0.05$). (**D**) Relative elastic recovery of skin tissue reconstructed with CEAs. The difference in mean values was statistically significant (i.e., "*", $p < 0.05$). Relative elastic recovery was substantially higher in the CEA-treated skin on the dorsal aspect of the forearm, indicating stiffer skin elasticity. Interestingly, when comparing the palmar and dorsal sites in both forearms, the skin reconstructed with Integra + CDEAs showed differential remodeling between the palmar and dorsal aspects of the forearm, not unlike that seen in unaffected, anatomically differentiated skin. This specific difference was about 4 times smaller in value between the palmar and dorsal sites treated with CEAs. This finding indicated that skin reconstructed with complex cutaneous grafts (i.e., recapitulating dermal and epidermal properties) may not only result in improved esthetics, but also undergoes superior functional remodeling over time. CEA, cultured epithelial autografts; CDEA, cultured dermo-epidermal autografts; TBSA, total body surface area.

2. Supplementary Tables

Table S1. Technical benchmarking (i.e., general manufacturing technical specifications) of the autologous and allogeneic cutaneous cell therapies implemented at the Lausanne University Hospital Burn Center. CEA, cultured epithelial autografts; CDEA, cultured dermo-epidermal autografts; GMP, good manufacturing practices; PBB, progenitor biological bandages.

Bioengineered Cutaneous Graft Type	PBB	CEA	CDEA
Type of Approach	Allogeneic	Autologous	Autologous
Cell Origin	GMP cell stock	Patient epidermal biopsy	Patient epidermal & dermal biopsy
Type of Cells	FE002-SK2 primary progenitor fibroblasts	Primary keratinocytes	Primary fibroblasts & keratinocytes
Minimal Time for Clinical Lot Preparation	24 hours	22.9 ± 4.2 days [1]	50.0 ± 8.5 days [2]
Standard Surface of Clinical Lots (m^2)	0.400	0.375	0.300
Frequency of Applications	Every 2–3 days	Every week	Every 1–2 weeks
Standard Number of Applications (n)	4	1–5	1–2

[1] Mean data gathered from 12 patient files and manufacturing batch records. [2] Mean data gathered from 3 patient files and manufacturing batch records.

Table S2. Statistical analysis results relative to the quantitative determination of collagen production levels in vitamin C stimulation assays, as reported in Figure 1. The results were expressed in the form of experimental *p*-values. Statistically significant differences were identified using an asterisk (i.e., "*") in the table where *p*-values were found to be <0.05. The results revealed that vitamin C stimulation resulted in significant induction of collagen production in all conditions and that the scale of the induction was greater in the keratinocyte proliferation medium for all of the considered cell types. CTRL, control; ns, non-significant.

**Primary Progenitor Fibroblasts**			
**Medium Composition**	**Vitamin C Stimulation** (Yes / No)	**Day 7 vs. Day 0** (*p*-value)	**Statistical Significance** (* / ns)
Fibroblast Medium	No (CTRL group)	0.0145	*
Fibroblast Medium	Yes	0.0030	*
Keratinocyte Medium	No (CTRL group)	0.0332	*
Keratinocyte Medium	Yes	0.0002	*

**Primary Patient Fibroblasts**			
**Medium Composition**	**Vitamin C Stimulation** (Yes / No)	**Day 7 vs. Day 0** (*p*-value)	**Statistical Significance** (* / ns)
Fibroblast Medium	No (CTRL group)	0.0997	ns
Fibroblast Medium	Yes	0.0355	*
Keratinocyte Medium	No (CTRL group)	0.0034	*
Keratinocyte Medium	Yes	0.0003	*
